# Preference of primary care patients for home-based healthcare and support services: a discrete choice experiment in China

**DOI:** 10.3389/fpubh.2024.1324776

**Published:** 2024-04-18

**Authors:** Yaqing Liu, Sixian Du, Chaojie Liu, Tianqin Xue, Yuqing Tang

**Affiliations:** ^1^School of Medicine and Health Management, Huazhong University of Science and Technology, Wuhan, Hubei, China; ^2^Department of Public Health, La Trobe University, Melbourne, VIC, Australia; ^3^Key Research Institute of Humanities and Social Sciences of Hubei Provincial Department of Education, Wuhan, Hubei, China

**Keywords:** discrete choice experiment, home-based healthcare and support services, willingness to pay, China, homecare

## Abstract

**Importance:**

This research, utilizing discrete choice experiments, examines the preferences and willingness to pay for home-based healthcare and support services among residents in China, a country grappling with severe aging population, an area often underexplored in international scholarship.

**Objectives:**

This study aims to solicit the preferences of primary care patients for home-based healthcare and support services in China.

**Design, setting, and participants:**

A discrete choice experiment (DCE) was conducted on 312 primary care patients recruited from 13 community health centers in Wuhan and Kunming between January and May 2023. The experimental choice sets were generated using NGene, covering five attributes: Scope of services, health professionals, institutions, insurance reimbursements, and visiting fees.

**Main outcomes and measures:**

The choice sets were further divided into three blocks, and each participant was asked to complete one block containing 12 choice tasks. Mixed logit models were established to estimate the relevant importance coefficients of and willingness to pay for different choices, while Latent Class Logit (LCL) modeling was conducted to capture possible preferences heterogeneity.

**Results:**

The relevant importance of the scope of services reached 67.33%, compared with 19.84% for service institutions and 12.42% for health professionals. Overall, respondents preferred physician-led diagnostic and treatment services. LCL categorized the respondents into three groups: Group one (60.20%) was most concerned about the scope of services, prioritizing disease diagnosis and treatment over preventive care and mental health, while group two (16.60%) was most concerned about care providers (hospitals and medical doctors were preferred), and group three (23.20%) was most concerned about financial burdens.

**Conclusion:**

Primary care patients prefer physical health and medical interventions for home-based healthcare and support services. However, heterogeneity in preferences is evident, indicating potential disparities in healthcare and support at home services in China.

## 1 Introduction

Population aging has emerged as a significant public health challenge, putting healthcare systems under considerable stress. According to projections, by 2030, one in six individuals worldwide will be aged over 60 years (1). This extended life expectancy has resulted in a growing population of individuals living with chronic conditions and disabilities (2). Such demographic and epidemiological transition has led to heightened disease prevalence and increased economic burdens. The mobility challenges faced by older adult or disabled individuals in accessing medical facilities highlight the significance of home-based healthcare and support services, enabling patients to receive treatment, care, or rehabilitation in the comfort of their own homes. According to China's State Council, ~90% of the older adult reside at home. As China becomes one of the countries with the largest older adult population in the world, the demand for such home-based services is steadily increasing.

Home-based healthcare and support services (HHSS) present a strategic approach to enhancing efficiency and maintaining care for those with the greatest needs (3–5). HHSS encompass a comprehensive range of care services, spanning from preventive measures to acute and post-acute care, administered by various institutions and healthcare professionals (6–8). Acute care ranging from emergency assistance for chronic diseases, referrals to specialized hospitals, to advanced in-home healthcare. Post-acute care encompasses home monitoring, management post-discharge, and rehabilitation physiotherapy, ensuring a seamless transition and continuous support from hospital to home. The notion of “hospital at home” was initially pioneered in the United Kingdom and subsequently adopted by the United States (9), Canada (10), Japan (11), and Australia (12). In the United States and the Netherlands, both hospitals and nursing homes offer hospital at home programs, serving as cost-effective alternatives to hospital-based acute and sub-acute care options (13–15). HHSS demonstrate superior accessibility, convenience, and efficiency compared to hospital-based services (5, 16), particularly among older patients (17). Previous research indicates that home-based treatment can serve as a cost-effective substitute for hospital care in conditions such as cellulitis (18), chronic obstructive pulmonary disease (COPD) (19), and ophthalmic diseases (20). This approach aids in alleviating the financial strain of chronic conditions on individuals and society at large (21, 22).

Discrete choice experiments (DCEs), grounded in the principles of random utility theory, represent a methodological paradigm originating from the field of economics and have found extensive application in health services research to scrutinize individual preferences (23). The conceptual underpinnings of DCEs are notably influenced by Lancaster and Rosen's attribute theories, particularly Lancaster's consumer demand theory, which underscores the pivotal role of attributes in shaping preferences (24–26). DCEs operationalize these theoretical foundations by presenting respondents with hypothetical scenarios, each delineated by diverse attributes, thereby eliciting choices among alternatives. This methodological design facilitates the systematic quantification of preferences, illuminating the inherent trade-offs individuals navigate in health service decision-making. In the healthcare domain, HHSS are delivered by healthcare professionals within patients' homes and comprise diverse attributes, such as service content, staff and providers, Medicare reimbursement rates, and reimbursement prices. These attributes collectively hold substantial influence over patient preferences. Notwithstanding the advantages, apprehensions regarding the safety and quality of HHSS persist (27). The home environment lacks the comprehensive infrastructure of hospitals (27), thereby constraining the range of services that can be provided. A recent study brought to light that insufficient consideration of psychosocial challenges within HHSS could compromise the quality of patient care (28). Moreover, the services eligible for health insurance reimbursement in the context of HHSS may be subject to limitations (29, 30), resulting in substantial out-of-pocket expenses (31).

In China, the responsibility for delivering HHSS falls upon community health centers (CHCs). Both hospitals and CHCs have the capacity to establish “home beds,” with designated healthcare professionals providing in-home services (32). Despite a history of HHSS in China dating back to 1984, the coverage and extent of HHSS have remained limited. Recognizing the fact that the country harbors the world's largest population living with chronic conditions and disabilities (33), the Chinese government acknowledges the substantial requirement for HHSS (34–36). Over the past 5 years, there has been a notable commitment from the Chinese government to advance HHSS, as evidenced by collaborative endeavors involving the State Council, the National Health Commission, as well as provincial and municipal administrations (37). Nonetheless, the adoption of HHSS has primarily occurred in the more developed regions, such as Beijing, Guangzhou, and Shenzhen. Inhabitants of less developed central and western areas have experienced a lesser degree of HHSS accessibility due to resource limitations, despite expressing higher satisfaction levels with HHSS in comparison to their wealthier counterparts in the eastern regions (38–40).

Our comprehension of patient preferences for HHSS remains quite limited. While a small number of studies have delved into patient preferences for hospital at home services, these investigations were carried out in developed countries with established universal health coverage (41, 42). Nevertheless, in developing nations, the financial burden remains a significant factor influencing patient decisions about care options (43). Furthermore, the delivery of HHSS can involve both formal and informal care providers (31). Hence, the aim of this study is to bridge this gap in the literature by employing a discrete choice experiment (DCE) (44) focused on primary care patients, aiming to ascertain their preferences for HHSS. Owing to limitations in time and resources, the study opted for Wuhan and Kunming as its focal points. These cities, situated in the central and western regions of China, respectively, are characterized by their dense populations and significant older adult demographics, making them ideal for the research.

## 2 Methods

### 2.1 Study settings

Employing purposive sampling, the study focused on Wuhan and Kunming, provincial capitals of Hubei (central China) and Yunnan (western China) respectively, chosen for their large populations and significant older adult demographics. Wuhan, the capital of Hubei province, stands as a significant inland transportation hub recognized for its well-developed manufacturing, automotive, and high-tech industries. It boasts a population of 13.65 million and an annual GDP of 177.1676 billion RMB, ranking 9th among all cities in mainland China (45). Approximately 11.81% of Wuhan's population is aged 65 years or older. On the other hand, Kunming, the capital of Yunnan province, serves as a prominent tourist destination. With an annual GDP of 722.250 billion RMB, it holds the 21st rank among all cities in mainland China. Kunming's population reaches 8.46 million, 10.49% of its residents aged 65 years or older (46, 47).

### 2.2 DCE design

We conducted a comprehensive review of governmental policy documents to discern the fundamental attributes linked with HHSS in mainland China. Utilizing keywords such as “home health service,” “long-term care support,” “hospital at home,” and “home bed,” we scoured the official websites of the State Council, the National Health Commission, the National Health Insurance Bureau, and their corresponding provincial counterparts. Our search encompassed policy documents released from January 1984 (marking the inception of HHSS in mainland China) up to June 2023. This effort yielded a total of 63 policy documents deemed relevant (Appendix A). Employing NVivo (QSR International Pty Ltd, Melbourne, Australia; version 11), we conducted content analysis on these documents. This analytical process entailed open coding, leading to the identification of eight key themes (Appendix A). From these themes, the research team selected five that are most likely to elicit concerns from patients for the purpose of designing the DCE. This selection was made based on the consideration that employing more than five attributes could overload cognitive capacity, potentially compromising the reliability of DCE outcomes (24, 48).

By presenting various combinations of these attributes and their options, the DCE empowers respondents to make selections (24). Consequently, it offers valuable insights into the genuine service requirements of residents and facilitates the refinement of service delivery strategies for both providers and governmental bodies (25). Table 1 presents the definitions and available options pertaining to the five attributes: Scope of services, health professionals, institutions, insurance reimbursements, and visiting fees. Effects coding was employed due to the predominantly qualitative character of the attribute options.

**Table 1:** Attributes associated with home-based healthcare and support services.

**Attributes**	**Option and coding**	**Definition**
Scope of services	0 = Health monitoring and management	Continuous in-home health monitoring entails the systematic tracking and analysis of individuals' data, including parameters like body temperature, blood pressure, blood sugar levels, and electrocardiograms. The primary aim is to identify and predict potential health issues (49, 50). Subsequent to data analysis, individuals receive health education and medical advice tailored to their needs. Additionally, health monitoring and management programs contribute to data compilation, thereby facilitating the identification of health concerns across the population.
	1 = Diagnosis and treatment	Diagnosis and treatment encompass acute care, sub-acute care, and chronic care management, involving activities such as consultations, examinations, diagnostics, and the provision of medical, nursing, and pharmacy services.
	2 = Care support services	Home delivery of meals, assistance with bathing, and the elimination of environmental obstacles to foster independent living.
	3 = Mental health services	Psychological counseling, spiritual comfort and palliative care for terminal illness.
Health professional	0 = Doctor	Registered doctors, including assistant doctors and rural health workers (village doctors).
	1 = Nurse	Registered nurses.
	2 = Others	Other skilled health workers such as rehabilitation therapists, clinical nutritionists, and pharmacists.
Institutions	0 = Hospitals	Hospitals include both public hospitals and for-profit private hospitals.
	1 = Primary care institutions	Primary care institutions include urban CHCs and rural township health centers.
	2 = Other institutions	Other institutions mainly consist of aged care facilities.
Insurance reimbursement	0 = 85%	Reimbursement of fees for services rendered at home, with the remainder of the fee being covered by patients through out-of-pocket payments.
	1 = 80%	
	2 = 75%	
Visiting fee	0 = 50 RMB per visit	The visiting fee encompasses transportation expenses and the associated costs related to human resources (51).
	1 = 65 RMB per visit	
	2 = 80 RMB per visit	

It should be emphasized that the providers of Home Health and Support Services (HHSS) in China encompass a variety of institutions, each with unique attributes in the provision of home-based healthcare and support. These include hospitals, primary medical and health institutions, nursing homes, and older adult care facilities (52). Hospitals tend to charge higher prices for their services, reflecting their emphasis on inpatient care. In contrast, primary medical and health institutions offer a more cost-effective model, delivering services directly to patients' homes through teams of family doctors. Meanwhile, nursing homes and older adult care facilities primarily operate by providing services within their facilities (53, 54). Consequently, the diverse nature of these HHSS institutions is considered a critical factor in the design of our discrete choice experiment (DCE).

Before finalizing the DCE design, an identical design was implemented as a research pilot, assuming minimal prior parameters derived from previous studies (55). The pilot involved 50 respondents with characteristics akin to the target population, sourced from two primary healthcare facilities in Wuhan, Hubei Province—a community ultimately excluded from formal data collection. To assess internal consistency, each respondent in the pilot encountered a selection set with one option consistently superior across all attribute levels. The pilot aimed to establish prior parameters for the final design, validate the questionnaire's option sets, and evaluate task comprehension. All pilot respondents exhibited internal conformance and encountered no cognitive difficulties in understanding the selection exercises.

Using the parameters obtained from the pilot study, we constructed a D-efficient design to improve the accuracy of the estimated parameters. The final experimental choice sets were generated using NGene (University of Copenhagen, Copenhagen, Denmark; version 1.2). The syntax crafted for input into NGene (Appendix B) resulted in the creation of 36 choice tasks. These tasks were designed to achieve a high level of D-optimality, reaching 98.47%, and a correction matrix of zero (C matrix). To mitigate the cognitive load on respondents, the choice tasks were distributed across three blocks, each containing 12 choice sets.

### 2.3 Target population and sample size

The target demographic for this study was defined as follows: (1) individuals residing in Wuhan, Hubei Province, or Kunming, Yunnan Province, for a period exceeding 6 months; and (2) individuals who, devoid of any cognitive impairments, expressed a willingness to participate fully in the questionnaire process. The necessary sample size was established using a rule of thumb formula for DCE studies suggested by Orem (56). Despite the adequacy of a sample size of 83 for the present DCE study, we augmented the size in anticipation of subgroup analysis necessitated by preference heterogeneity.

In our approach to discrete choice experiments (DCEs), we intentionally did not include an opt-out option, a decision that, despite its limitations, offers significant advantages in accurately reflecting real-world decision-making contexts. In scenarios where individuals face mandatory choices, such as in decisions regarding essential services or treatments, the exclusion of an opt-out option in discrete choice experiments (DCEs) can more accurately mirror the real-world decision-making context (57), thereby enhancing the validity of research findings. This approach ensures that respondents' selections are confined to the presented alternatives, facilitating a direct comparison between new and existing interventions or products (58). However, this methodological choice carries implications for the accuracy of research outcomes. Firstly, incorrect specification of the opt-out effect may compromise the precision in estimating marginal willingness to pay and in predicting market share, potentially undermining the validity of research conclusions (59). Secondly, the absence of an opt-out option may overlook the preferences of non-demanders, who would not select any of the available options (60).

### 2.4 Data collection

**Figure 1:**
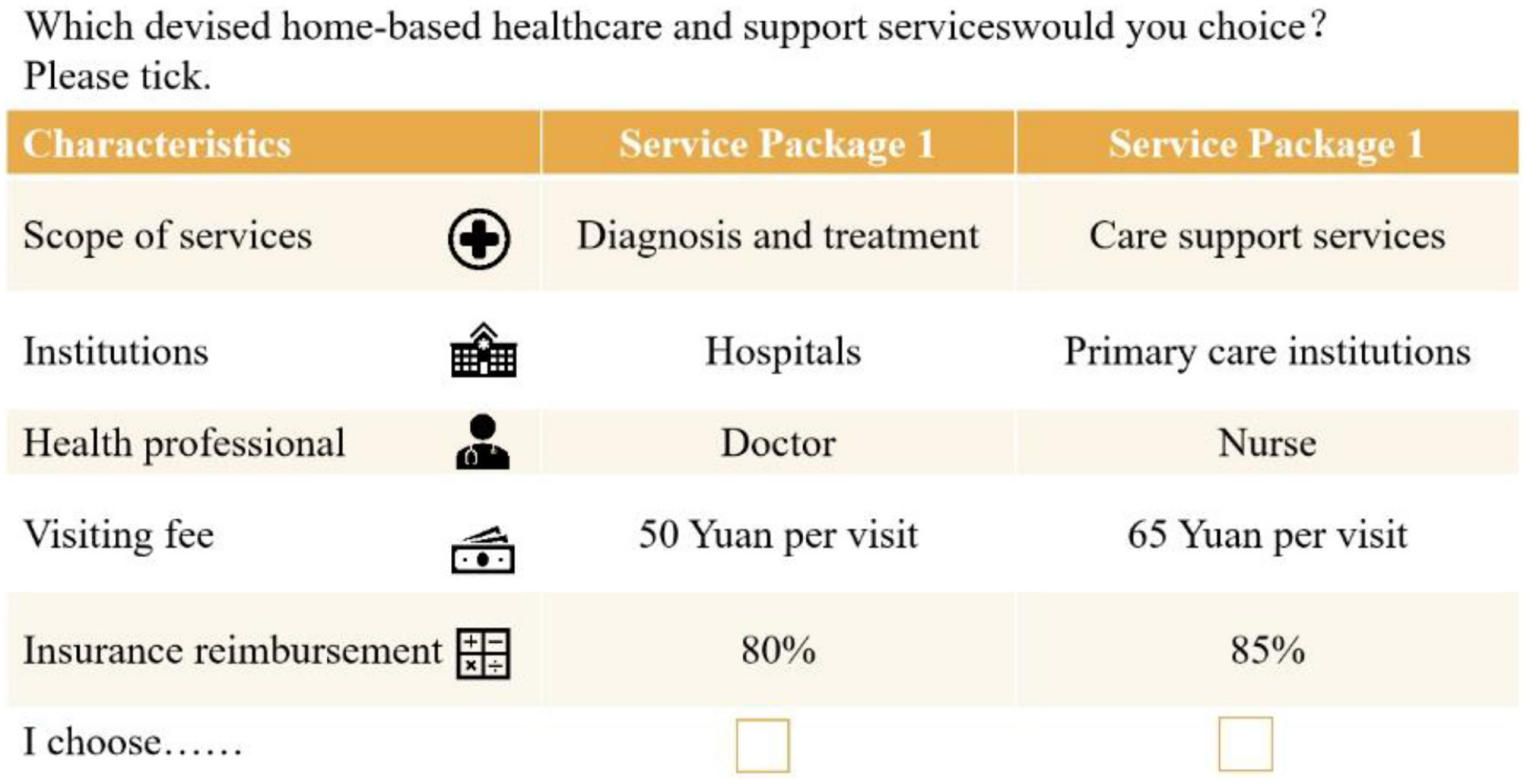
An example of choice tasks.

A cross-sectional questionnaire based on a face-to-face survey was used, and eligible affected respondents were recruited from primary health care organizations in Wuhan, Hubei Province, China, and Kunming, Yunnan Province, China. We also provided a budget reminder to ensure that the willingness-to-pay (WTP) estimates for the discrete choice experiment were accurate when we administered the questionnaire to respondents face-to-face (61). Data collection was conducted through a structured questionnaire (Appendix D), which was organized into four distinct sections: the socioeconomic status of participants, their previous encounters with HHSS, the forced choice tasks, and an assessment of personal wellbeing. Participants were presented with two scenarios in each choice task, each characterized by the five attributes (refer to example in Figure 1). To ensure the reliability of responses, one choice set was duplicated to assess the consistency of participants' selections.

The necessary sample size was established using a rule of thumb formula for DCE studies suggested by Orme (56). Despite the adequacy of a sample size of 83 for the present DCE study, we augmented the size in anticipation of subgroup analysis necessitated by preference heterogeneity. Overall, a total of 387 questionnaires were successfully filled out, and 312 (80.6%) of these were deemed valid (171 from Wuhan and 141 from Kunming) for further analysis after excluding 75 responses that did not meet the consistency test criteria.

### 2.5 Criteria for inclusion and exclusion

We have designed inclusion and exclusion criteria to ensure the integrity of data collected via DCE questionnaires. For inclusion, participants must complete the questionnaire in its entirety and show consistent choices across repeated choice sets, confirming the reliability of their responses. Questionnaires with any missing answers or inconsistencies in these repeated sets will be excluded, as such discrepancies may signal misunderstandings or insufficient engagement. These criteria are fundamental for upholding the study's methodological integrity, by ensuring that included responses are characterized by diligence and consistency, thus bolstering the overall accuracy and trustworthiness of the research findings. Overall, a total of 327 questionnaires were successfully filled out, and 312 (95.41%) of these were deemed valid (171 from Wuhan and 141 from Kunming) for further analysis after excluding 75 responses that did not meet the consistency test criteria.

### 2.6 Statistical analysis

The main effects were calculated using an optimal orthogonal design based on the differences assumption with zero priors (62, 63). A Mixed Logit model (MIXL) was constructed, a choice favored by the literature for comprehensive population preference analysis on a large scale (64–66). As expressed in Equation (1), the utility *U*_*mni*_ derived by an individual m from scenario *i* in a choice set *n* is composed of the systematic component *V*_*mni*_ and the random utility component εε_*mni*_.


(1) $$\begin{array}{l} U_{mni}=V_{mni}+\varepsilon_{mni\;}=\beta^{{\;}^{\prime }}X_{mni}+\varepsilon_{mni\;} \end{array}$$

Where β′ represents a vector of relevant preference parameters, and *X*_*mni*_ signifies a vector encompassing attributes of the alternative scenarios.

Given that our DCE design encompasses five attributes, the utility function for individual m's selection of scenario i can be portrayed through Equation (2), wherein each β-parameter corresponds to a distinct attribute, and all β-parameters adhere to a random normal distribution. The term ε_mni_ denotes the error component.


(2) $$\begin{array}{l} U_{mni}=\beta_{1m}Scope_{mni}+\beta_{2m}Institution_{mni}+\beta_{3m}Professional_{mni}\\ +\beta_{4m}Visiting\;Fee_{mni}+\beta_{5m}Insurance\;Reimbursement_{mni}\\ +\varepsilon_{mni} \end{array}$$

To gauge the relative importance of each attribute, we computed the ratio of the disparity between the highest and lowest β coefficient values for each attribute to the sum of the disparities between the highest and lowest β coefficient values for all attributes (56, 67).

We further calculated the Willingness to Pay (WTP) by dividing the negative coefficients of the non-monetary attributes by the coefficient of the monetary attribute “visiting fee,” as outlined in Equation (3). These WTP values provide a more easily comprehensible insight into the relative significance respondents attached to the pertinent DCE attributes in comparison to the β coefficients.


(3) $$\begin{array}{l} WTP_{a}=-\left(\frac{\beta_{a}}{\beta_{Visiting\;Fee}}\right) \end{array}$$

To account for potential variations in preferences, we employed Latent Class Logit (LCL) models (68–70). These models were designed to capture heterogeneity among respondents, considering factors like gender, age, educational background, and prior experience with HHSS. The LCL models classify participants into distinct groups by utilizing metrics such as the Bayesian information criterion and the chi-square information criterion (71, 72).

All data analyses were conducted using STATA (StataCorp LLC, College Station, USA; version 16). In our study, we constructed STATA commands for mixed logit model (73) and latent logit model (74). For further commands and detailed specifications used in our analysis, readers are directed to the Supplementary material. This approach ensures that our methodology is both transparent and replicable, allowing for a deeper understanding and potential replication of our study's findings. A two-tail *p*-value < 0.05 was considered statistically significant.

## 3 Results

### 3.1 Description of the study population

A total of 327 respondents partook in the experiment, with 312 successfully passing the consistency test, with a success rate of 95.41% (Table 2). A significant portion of the final respondents comprised older adult individuals with chronic health conditions, representing potential recipients of HHSS in Kunming, Hubei, and Yunnan, China. Slightly more than half (54.49%) of the study participants were women. The respondents had an average age of 42.61 years (SD = 11.32), with 35.90% falling into the 60 years or older category. The majority held a university degree (54.49%), lived in urban areas (83.65%), and had no prior experience with HHSS (63.14%). It was observed that they were most commonly part of households consisting of three members (46.15%). In the study population, 32.37% had no chronic diseases, 48.40% had one, and 19.23% reported two or more. Prevalent chronic diseases included cardiovascular and cerebrovascular diseases (41.99%), endocrine diseases (28.21%), respiratory diseases (19.23%), and cancers or other diseases (4.81%). The data outlines that 64.42% of individuals have medical insurance for urban workers, 29.49% have insurance for both urban and rural residents, 3.85% hold commercial medical insurance like major disease coverage, and 2.24% lack any medical insurance.

**Table 2:** Characteristics of study participants (*n* = 312).

**Characteristics**	***n***	**%**
**Sex**		
Male	142	45.51
Female	170	54.49
**Age (years)**		
≤ 44	172	55.13
45–59	28	8.97
60–74	20	6.41
75–89	89	28.53
≥90	3	0.96
**Educational attainment**		
Junior high school and below	71	22.76
High school or vocational college	25	8.01
Associate degree	46	14.74
Bachelor's degree	124	39.74
Master's degree	37	11.86
Doctoral degree	9	2.88
**Monthly household income after tax (RMB)**		
3,001–5,000	42	13.46
5,001–10,000	139	44.55
10,001–15,000	67	21.47
15,001–20,000	31	9.94
>20,000	33	10.58
**Residence**		
Urban	261	83.65
Urban-rural joint	23	7.37
Rural	28	8.97
**Number of household members in the past year**		
≤ 2	64	20.52
3	144	46.15
4	75	24.04
≥5	29	9.29
**Use of home-based healthcare and support services**		
Never	197	63.14
Yes	115	36.86
**Number of chronic diseases**		
0	101	32.37
1	151	48.40
≥2	60	19.23
**Types of chronic diseases**		
Cardiovascular and cerebrovascular diseases (hypertension, coronary heart disease, etc.)	131	41.99
Endocrine diseases (diabetes, etc.)	88	28.21
Respiratory diseases (COPD, bronchitis, etc.)	60	19.23
Cancers or other diseases	15	4.81
**Types of medical insurance purchased**		
Medical insurance for urban workers	201	64.42
Medical Insurance for urban and rural residents	92	29.49
Commercial medical insurance such as major disease insurance	12	3.85
No medical insurance	7	2.24

### 3.2 Mix logit model and WTP space

Table 3 presents the results of the fundamental panel MIXL model and WTP Space. The mean coefficients for each attribute in the main effects panel MIXL model exhibit the hypothesized signs. The chi-square test for model fit indicates that the basic panel MIXL model is statistically significant (*P* < 0.05).

**Table 3:** Main effects panel MIXL model preference weights and WTP space.

**Attributes**	**Option**	**Preference estimates**				**WTP space**	
		**Mean**	**SD**	**Relative importance (%)**	**Order of importance**	**Mean**	**SD**
		**β**	**β**			**β**	**β**
Scope of services (ref: health monitoring and management)	Diagnosis and treatment	0.100	0.397^**^	-	-	−0.714	−40.060^***^
	Care support	−0.436^***^	−0.014	13.491	3	−47.226^***^	−14.586
	Mental health services	−1.607^***^	1.443^***^	49.762	1	−161.495^***^	−131.822^***^
Health professional (ref: physicians)	Nurses	−0.232^***^	0.046	7.188	5	−25.106^**^	−22.877^*^
	Others	−0.547^***^	−0.577^***^	16.943	2	−54.597^***^	53.862^***^
Institutions (ref: hospitals)	Primary care institutions	0.106	0.000	–	-	7.733	−5.652
	Others	−0.397^***^	−0.019	12.296	4	−36.991^***^	4.969
Insurance reimbursement rate (fixed)		0.815	−4.439^**^	-	-	45.521	25.139
Visiting fee		−0.010^***^	0.026^***^	0.321	6	-	-
Numbers of observations		7,488.000				7,488.000	
Numbers of respondents		312.000				312.000	
Wald χ^2^		211.660				1,499.740	
*P*>χ2		0.000^***^				0.000^***^	
Log pseudo-likelihood		−2,220.835				−2,223.287	
Akaike information criterion		4,459.669				4,464.575	
Bayesian information criterion		4,521.958				4,526.864	

SD, Standard Deviation.

WTP space were in RMB. US $1 = 7.15 RMB.

^*^P < 0.05; ^**^P < 0.01; ^***^P < 0.001.

The relative importance of an attribute is calculated by dividing the range of that attribute by the total sum of ranges for all attributes (74). According to Table 3, in terms of preference impact among respondents for HHSS services, the ranking of attribute importance is as follows: Mental Health Services (49.762%), Other Health Professionals (16.943%), and Care Support (13.491%).

The study results show that respondents have a higher preference for HHS services that involve health monitoring and management, diagnosis and treatment, are provided by physicians or hospitals, and have a visiting fee. In terms of service content, compared to health monitoring and management services, respondents significantly prefer mental health services less. The fixed insurance reimbursement rate does not significantly influence respondents' preferences for HHSS.

Statistically significant Standard Deviation estimates in the effects panel MIXL model indicate consistency in preferences for the attributes of Scope of Services, Health Professional, Insurance Reimbursement Rate, and Visiting Fee. Additionally, we used the LCL model to generate preference estimates for HHSS (Table 4). The log-likelihood ratio test further confirms that the LCL model fits better than the panel MIXL model (*P* < 0.001).

**Table 4:** Results of the latent class logit model.

**Attributes**	**Option**	**Group 1 (*****n*** = **188)**			**Group 2 (*****n*** = **52)**			**Group 3 (*****n*** = **72)**		
		**β**	**SE**	**WTP**	**β**	**SE**	**WTP**	**β**	**SE**	**WTP**
Scope of services (ref: health monitoring and management)	Diagnosis and treatment	0.121	0.079	15.005	0.032	0.210	−2.509	0.001	0.136	0.030
	Care support	−0.892^***^	0.145	−110.707^*^	0.104	0.259	−8.102	0.456^*^	0.200	24.279^**^
	Mental health services	−2.379^***^	0.162	−295.456^**^	−0.669^*^	0.262	52.243	0.496^**^	0.178	26.432^**^
Health professional (ref: physicians)	Nurses	−0.277^**^	0.095	18.929	−0.916^***^	0.206	34.700^*^	0.362^*^	0.150	4.593
	Others	−0.328^**^	0.096	−23.704	−2.459^***^	0.336	86.853^*^	−0.009	0.138	−12.303
Service institution (ref: hospitals)	Primary care institutions	0.152	0.089	34.382^*^	−0.445	0.228	71.535	0.086	0.125	19.271
	Others	−0.191^**^	0.083	−40.751	−1.113^***^	0.210	191.945^*^	0.125	0.131	−0.493
Visiting fee		−0.008^**^	0.003	–	0.013	0.007	–	−0.019^***^	0.004	-
Insurance reimbursement rate (fixed)		−1.740^*^	0.847	−216.048	0.698	1.893	−54.483	5.156^***^	1.386	274.737^*^
Share of respondents (%)	60.200			16.600			23.200		
Female gender	0.388			0.893			0.000		
≥60 year of age	1.648			−13.707			0.000		
University degree	0.616			2.312			0.000		
Past use of home-based healthcare and support services	1.526			1.903			0.000		
Constant	−0.491			−2.777			0.000		
Log pseudo-likelihood	−2,137.778								
Akaike information criterion	4,349.555								
Bayesian information criterion	4,488.046								
Conditional Akaike information criterion	4,525.046								

SE, Standard error.

WTP space were in RMB. US $1 = 7.15 RMB.

^*^P < 0.05; ^**^P < 0.01; ^***^P < 0.001.

We estimate respondents' willingness to pay (WTP) for HHSS based on the Visiting Fee. For non-linear variables with effect coding, the base level of utility coefficients is the negative sum of other levels, and the WTP to move from one level of an attribute to another is the difference in the corresponding coefficients (75). Regarding the Scope of Service, compared to health monitoring and management, respondents are willing to pay 47.226 RMB less for Care Support services and 161.495 less for Mental Health Services. In terms of Health Professionals, compared to physicians, Nurses are willing to pay 25.106 RMB less, and others are willing to pay 54.597 RMB less. For Institutions, compared to hospitals, respondents' willingness to pay decreases by 36.991 RMB for others. Notably, changes in the Insurance Reimbursement Rate did not significantly affect respondents' willingness to pay, which may relate to the small range of settings for the Insurance Reimbursement Rate.

### 3.3 Latent Class Logit model

The Latent Class Logit model revealed the presence of three latent class groups (Appendix D, Table 4).

#### 3.3.1 Group 1: service-oriented majority

Group 1, making up the majority at 60.20%, prioritizes immediate healthcare interventions, such as disease diagnosis and treatment, over preventive care and mental health services. Their preferences suggest a reactive healthcare approach, focused on addressing acute health issues as they arise rather than engaging in preventative strategies or mental health considerations.

#### 3.3.2 Group 2: provider-selective segment

Comprising 16.60% of respondents, Group 2 values professional qualifications and the setting of care, with a clear preference for hospitals and physicians over other healthcare providers. This group tends to be younger and more educated, which may influence their trust in formal medical institutions and expectations of healthcare providers. The insignificance of the visiting fee in group 2 may be due to the fact that members of group 2 are older than those in the other two groups, making them less sensitive to the price of HHSS, while having higher expectations of service providers.

#### 3.3.3 Group 3: financially-concerned respondents

At 23.20%, Group 3 is characterized by a strong concern for the financial aspects of healthcare, such as insurance reimbursement and visiting fees. Their reluctance to utilize home-based healthcare services suggests that cost is a significant factor in their healthcare decisions, highlighting the need for more affordable options or financial support for accessing services.

The Willingness to Pay (WTP) metric in the table represents the visiting fee that respondents in each group would be willing to pay for the specified healthcare services (Table 4). For Group 1, the WTP is negative for “Care support” (−110.707 RMB) and “Mental health services” (−295.456 RMB), suggesting that respondents are less inclined to pay for these services compared to the baseline “Health monitoring and management.” Conversely, for “Mental health services” in Groups 2 and 3, the WTP values are positive (52.243 and 26.432 RMB, respectively), indicating a higher willingness to pay for these services over the baseline. The positive WTP for “Care support” in Group 3 (24.279 RMB) also signifies a higher valuation for this service by respondents. It should be noted that the insurance reimbursement rate for Group 3 shows a substantially higher WTP (274.737 RMB), reflecting a preference for higher insurance reimbursements.

**Figure 2:**
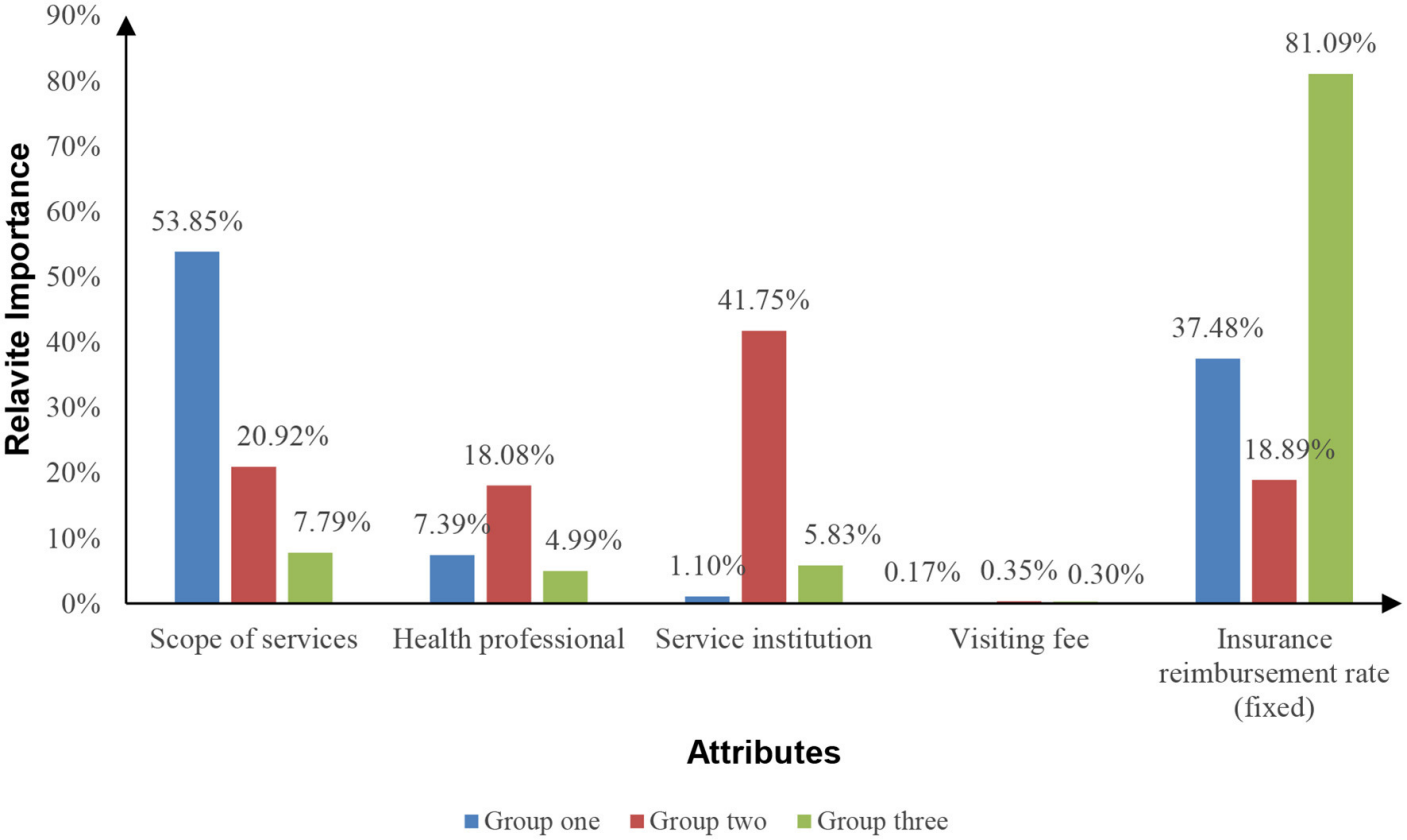
Relative importance of three group.

We assessed the relative importance of various attributes across three groups to examine how different attributes influence residents' preferences for HHSS. Among the identified groups, members of group one attributed the greatest relative importance to the scope of services (53.85%). Conversely, group two members accorded the highest relative importance to health professionals (41.75%) and service institutions (189.08%). Group three members exhibited the highest relative importance (81.09%) toward insurance reimbursement rates (Figure 2).

## 4 Discussion

This study is pioneering in its approach to eliciting the preferences of primary care patients for home-based healthcare and support services (HHSS) in China, utilizing a discrete choice experiment (DCE). Prior DCE studies have primarily concentrated on the preferences of individuals with specific conditions like dementia and COPD for hospital-at-home services, rather than addressing the comprehensive array of HHSS, typically in developed country settings (41, 42). In this research, we specifically investigated the preferences of primary care patients across five attributes: Scope of services, health professionals, service institutions, insurance reimbursement, and visiting fees. Notably, the scope of services emerged as the most pivotal factor, trailed by service institutions and service staff. There was a clear inclination toward physical health interventions and medical care delivered by physicians employed by healthcare facilities compared to alternative options. While a lower visiting fee was favored, no distinct preference was discerned for higher insurance reimbursement rates.

Undoubtedly, preventive care and medical interventions constitute significant factors when primary care patients opt for HHSS. These services predominantly cater to early interventions for minor acute ailments (75) and the ongoing management of chronic conditions. These findings echo a previous study, which emphasized the popularity of home medical visits and health education among older adults with disabilities (76). Empirical evidence supports the effectiveness of HHSS for patients with conditions like COPD and cardiovascular diseases, demonstrating outcomes comparable to hospitalization (77). Morton's study in Australia revealed that patients with chronic kidney disease were willing to sacrifice up to 23 months (95% CI, 19–27) of their life expectancy to undergo home dialysis, primarily to mitigate travel-related constraints (78). It is important to acknowledge that, despite the growing emphasis on strengthening integrated care for the older adult, especially those with chronic conditions, care support and mental health services do not seem to hold substantial interest among primary care patients for HHSS. However, numerous studies have underscored individuals' preference for receiving end-of-life care and mental health support at home (77, 79–81). The disparities in findings between our study and others might be attributed to cultural and contextual variations. The studies mentioned earlier were predominantly conducted in Western countries, whereas in China, mental health services and end-of-life care might still be subject to stigma within the general populace.

While many HHSS could be effectively administered by nurses, such as health monitoring and management, our study participants exhibited a preference for physicians. This inclination aligns with their preference for medical diagnosis and treatment, which might reflect the medical dominance prevalent in China's healthcare system (82). It is noteworthy that physicians indeed play a critical role in shaping the quality of medical services, a pivotal factor in people's decision-making concerning HHSS (83). However, it is crucial to acknowledge that patient care outcomes are the result of collaborative efforts from a variety of healthcare professionals. Nurses, in particular, play a vital role in executing medical advice, ensuring patient adherence, and cultivating a supportive environment for care. Numerous nursing services can be effectively provided in a patient's home setting (84). In certain contexts, pharmacy professionals can also contribute indispensably to HHSS (85). For instance, in Barcelona, geriatric Hospital at Home services are provided by a multidisciplinary team encompassing a geriatrician, two nurses, a physical therapist, an occupational therapist, and a social worker (86). Similarly, China emphasizes a team-based approach in HHSS, typically consisting of a physician, a nurse, and a public health worker (87).

In line with the observed preference for physical health and medical interventions administered by physicians, our study participants exhibited a preference for hospitals and primary care facilities as preferred institutions for HHSS. An intriguing finding was that participants in Group 1 of LCL, were willing to pay an additional 34.382 RMB for HHSS from primary care institutions compared to hospitals. This might reflect a sample bias, as our participants were drawn from those visiting primary care institutions. With the rapid evolution of technology, many inpatient hospital services have transitioned to Hospital at Home models (88, 89), particularly evident during the COVID-19 pandemic (89, 90). Empirical evidence supports the effectiveness of Hospital at Home in postoperative patient recovery and the management of complex conditions among older patients (91). In China, primary care institutions possess the capability to offer certain services that might only be available in hospitals in other countries. Given the congestion often witnessed in hospitals, primary care institutions could potentially be better positioned to provide HHSS. They are closely integrated with their communities and boast high levels of accessibility. In recent years, the Chinese government has endorsed collaborations and partnerships between hospitals and primary care institutions (92, 93) to enhance the efficiency and efficacy of patient care (94).

The findings from our study indicate that financial constraints do not pose a significant hurdle in the decision-making process of primary care patients regarding HHSS, despite their preference for a lower visiting fee. However, this observation is likely influenced by the comprehensive range of HHSS available, many of which are characterized by low-cost services. Additionally, the absence of a clear indication of the level of reimbursable medical expenses could play a role. In China, HHSS is conceived as a convenient and affordable care option for patients in need, which simultaneously helps alleviate the burden on healthcare resources (87). It is important to acknowledge that the visiting fee charged in HHSS is akin to the appointment fee for hospital outpatient visits. For individuals who receive recurring home visits, the cumulative financial strain over an extended duration cannot be dismissed. The latent class logit model developed in our study highlights that 23.20% of respondents were most concerned about financial burdens when selecting HHSS. This trend is not exclusive to China. In other contexts, Hospital at Home with reduced costs has been observed to attract inpatients as an alternative to traditional inpatient care (95).

### 4.1 Suggestions

Based on the findings of our research and considering the current landscape of Home Health Service Systems (HHSS) in China, we recommend several policy measures aimed at enhancing service supply and quality, improving affordability, and bolstering awareness and promotion of HHSS. These recommendations are crafted with a view to address the nuanced needs of residents, particularly those with chronic conditions and the older adult, drawing lessons from international best practices.

#### 4.1.1 Enhancement of service supply and quality

It is imperative for the Chinese government to focus on supply-side reforms in HHSS, with an expansion of service scope as a priority. Both the government and medical institutions should concentrate on providing comprehensive door-to-door diagnostic, treatment, and care support services, tailored to meet the specific needs of residents. Special attention should be directed toward vulnerable groups, including patients with chronic diseases and the older adult with significant age-related impairments. Drawing inspiration from Japan's Intensive Home Care Support Clinics (96) and the UK's emphasis on home hospice care for advanced cancer patients (97), these initiatives aim to reduce hospital admissions and facilitate the home management of chronic conditions, thus enhancing the overall quality of HHSS.

#### 4.1.2 Improvement in affordability

Financial considerations are a critical barrier to the widespread adoption of HHSS among Chinese residents. To address this, adjustments in health insurance reimbursement policies that positively impact home healthcare utilization should be made (98). Regions across central and western China should expand their health insurance coverage to include HHSS, following the examples set by Guangdong and Shanghai. Additionally, adopting Zhuhai's approach of offering direct financial assistance to economically disadvantaged individuals could further alleviate affordability concerns, making HHSS a viable alternative to traditional inpatient services.

#### 4.1.3 Enhancement of awareness and promotion

Our study indicates a lack of awareness and utilization of HHSS among the population. Governments, the CDC, hospitals, and primary healthcare organizations should intensify efforts to promote HHSS to match the growing demand. By emulating the promotional strategies of countries like Germany (99), the USA (100), which advocate for the adoption of smart home hospital beds and Internet-based services, China can enhance the efficiency and quality of HHSS. Such initiatives not only inform the public about the availability and benefits of HHSS but also encourage healthcare providers to integrate these innovative solutions into their service offerings.

Implementing these policy recommendations requires a concerted effort from all stakeholders involved in healthcare provision. By focusing on these strategic areas, China can significantly improve its HHSS infrastructure, making it more responsive to the needs of its residents, especially those requiring long-term care.

## 5 Limitations

The study is subject to several limitations that merit consideration. Firstly, due to time and budgetary constraints, the research was confined to two cities within western China, which may introduce a degree of sampling bias. As such, caution should be exercised when attempting to generalize the findings across a broader demographic. Secondly, the focus on the capital cities of two provinces in central and western China might have resulted in a sample with education and income levels above the national average, potentially causing a minor discrepancy in the assessment of residents' preferences and willingness to pay (WTP) for Home Health Service Systems (HHSS). Thirdly, the experimental design adopted in this study did not include an opt-out option, compelling respondents to make a choice and thus treating each participant as a “demander.” This approach may slightly distort the accuracy of the measured preferences for HHSS among residents (59). Fourthly, the study did not explore additional attributes that could influence patient preference. Quality, for instance, is a significant determinant of healthcare service selection and utilization (101). Previous research has shown the effectiveness of personalized care in promoting the adoption of Hospital at Home services, highlighting an area for potential future investigation. Lastly, while the research involved collecting data on the types of chronic diseases and the various forms of medical insurance held by participants, no correlation between these fundamental characteristics and residents' preferences for HHSS was identified at this stage. It is anticipated that subsequent studies will delve deeper into the relationship between these variables, thereby enriching our understanding of the factors influencing HHSS preferences.

## 6 Conclusions

Primary care patients in China have demonstrated a preference for physical health interventions and medical services when it comes to home-based healthcare and support services (HHSS). However, this preference reveals inherent heterogeneity, highlighting potential disparities in HHSS across the country. While addressing individual preferences is crucial, it is equally imperative for governmental initiatives to target the reduction of inequities in HHSS by minimizing financial barriers to access.

Primary care institutions are well-poised to effectively deliver HHSS, capitalizing on their skilled workforce and technological capacities. Simultaneously, HHSS should prioritize the reinforcement of health education and preventive care, which could contribute to enhancing the acceptability of such services. Adopting a team-based approach, preferably under physician leadership, is essential for effective HHSS delivery. Collaborative efforts among healthcare professionals not only optimize skill diversity but also help alleviate the high demand on physicians. Moreover, a team approach is pivotal in delivering personalized and tailored services, catering to the individual needs of patients.

## Data availability statement

The original contributions presented in the study are included in the article/Supplementary material, further inquiries can be directed to the corresponding author.

## Ethics statement

The studies involving humans were approved by Huazhong University of Science and Technology. The studies were conducted in accordance with the local legislation and institutional requirements. Written informed consent for participation in this study was provided by the participants' legal guardians/next of kin. The animal studies were approved by Huazhong University of Science and Technology. Written informed consent was obtained from the owners for the participation of their animals in this study. Written informed consent was obtained from the individual(s), and minor(s)' legal guardian/next of kin, for the publication of any potentially identifiable images or data included in this article.

## Author contributions

YL: Conceptualization, Data curation, Writing—original draft. SD: Conceptualization, Data curation, Formal analysis, Investigation, Resources, Software, Writing—original draft. CL: Formal analysis, Supervision, Visualization, Writing—review & editing. TX: Data curation, Software, Writing—original draft. YT: Conceptualization, Funding acquisition, Investigation, Resources, Visualization, Writing—original draft, Writing—review & editing.

## References

[1] World Health Organization. Ageing and Health. (2022). Available online at: https://www.who.int/zh/news-room/fact-sheets/detail/ageing-and-health# (accessed January 10, 2022).

[2] World Health Organization. Noncommunicable Diseases. (2022). Available online at: https://www.who.int/news-room/fact-sheets/detail/noncommunicable-diseases (accessed February 1, 2024).

[3] Umans VA van Ramshorst J de Boer S. Hospital at home. J Am Coll Cardiol. (2020) 75(11Suppl.1):3500. 10.1016/S0735-1097(20)34127-9

[4] Danielsson P Leff B. Hospital at home and emergence of the home hospitalist. J Hosp Med. (2019) 14:382–4. 10.12788/jhm.3162 30897054

[5] Burton LC Leff B Harper M Ghoshtagore I Steinwachs DA Greenough WB. Acceptability to patients of a home hospital. J Am Geriatr Soc. (1998) 46:605–9. 10.1111/j.1532-5415.1998.tb01077.x 9588374

[6] Gonçalves-Bradley DC Iliffe S Doll HA Broad J Gladman J Langhorne P. Early discharge hospital at home. Cochr Datab Syst Rev. (2017) 2017:CD000356. 10.1002/14651858.CD000356.pub4 PMC6481686 28651296

[7] Shepperd S Doll H Broad J Gladman J Iliffe S Langhorne P. Hospital at home early discharge. Cochrane Database Syst Rev. (2009) CD000356. 10.1002/14651858.CD000356.pub3 PMC4175532 19160179

[8] Singh R Rowan J Burton C Galletly C. How effective is a hospital at home service for people with acute mental illness? Austral Psychiatr. (2010) 18:512–6. 10.3109/10398562.2010.526214 21117838

[9] Patel HY West DJ Jr. Hospital at home: an evolving model for comprehensive healthcare. Glob J Qual Saf Healthc. (2021) 4:141–6. 10.36401/JQSH-21-4 37261225 PMC10229033

[10] Lapointe-Shaw L Jones A Ivers NM Rahim A Babe G Stall NM. Homebound status among older adult home care recipients in Ontario, Canada. J Am Geriatr Soc. (2022) 70:568–78. 10.1111/jgs.17501 34642950

[11] Morioka N Kashiwagi M Hamano J. Adherence to personal protective equipment use in home-care service agencies during COVID-19 in Japan: a cross-sectional survey. J Am Med Direct Assoc. (2022) 23:930–5.e2. 10.1016/j.jamda.2022.02.012 35337791 PMC8882398

[12] Palesy D Jakimowicz S Saunders C Lewis J. Home care in Australia: an integrative review. Home Health Care Serv Q. (2018) 37:113–39. 10.1080/01621424.2018.1438952 29424658

[13] Singh S Gray A Shepperd S Stott DJ Ellis G Hemsley A. Is comprehensive geriatric assessment hospital at home a cost-effective alternative to hospital admission for older people? Age Ageing. (2022) 51:afab220. 10.1093/ageing/afab220 34969074 PMC8753046

[14] Jones M Hill T Coupland C Kendrick D Akbari A Rodgers S. Cost-effectiveness of England's national 'Safe At Home' scheme for reducing hospital admissions for unintentional injury in children aged under 5. Inj Prev. (2023) 29:158–65. 10.1136/ip-2022-044698 36600567

[15] U ECY Pryor GA Parker MJ. Hospital at home - a review of our experience. Sicot J. (2017) 3:60. 10.1051/sicotj/2017047 29043966 PMC5646172

[16] Shepperd S Butler C Cradduck-Bamford A Ellis G Gray A Hemsley A. Is comprehensive geriatric assessment admission avoidance hospital at home an alternative to hospital admission for older persons? A randomized trial. Ann Intern Med. (2021) 174:889–98. 10.7326/M20-5688 33872045 PMC7612132

[17] Ross H Dritz R Morano B Lubetsky S Saenger P Seligman A. The unique role of the social worker within the Hospital at Home care delivery team. Soc Work Health Care. (2021) 60:354–68. 10.1080/00981389.2021.1894308 33645451

[18] Ibrahim LF Huang L Hopper SM Dalziel K Babl FE Bryant PA. Intravenous ceftriaxone at home versus intravenous flucloxacillin in hospital for children with cellulitis: a cost-effectiveness analysis. Lancet Infect Dis. (2019) 19:1101–8. 10.1016/S1473-3099(19)30288-9 31420292

[19] Criner GJ Dreher M Hart N Murphy P. COPD home oxygen therapy and home mechanical ventilation: improving admission-free survival in persistent hypercapnic COPD. Chest. (2018) 153:1499–500. 10.1016/j.chest.2018.03.053 29884255

[20] Liu Y Zheng Y Bressler NM. Express medicine-potential for home-based medical care. J Am Med Assoc Ophthalmol. (2021) 139:269–70. 10.1001/jamaophthalmol.2020.5981 33410918

[21] Federman AD Soones T DeCherrie LV Leff B Siu AL. Association of a bundled hospital-at-home and 30-day postacute transitional care program with clinical outcomes and patient experiences. J Am Med Assoc Intern Med. (2018) 178:1033–40. 10.1001/jamainternmed.2018.2562 29946693 PMC6143103

[22] Ausserhofer D Deschodt M De Geest S van Achterberg T Meyer G Verbeek H. “There's no place like home”: a scoping review on the impact of homelike residential care models on resident-, family-, and staff-related outcomes. J Am Med Dir Assoc. (2016) 17:685–93. 10.1016/j.jamda.2016.03.009 27130574

[23] Manski CF. Daniel McFadden and the econometric analysis of discrete choice. Scand J Econ. (2001) 103:241. 10.1111/1467-9442.00241

[24] Lancsar E Louviere J. Conducting discrete choice experiments to inform healthcare decision making: a user's guide. Pharmacoeconomics. (2008) 26:661–77. 10.2165/00019053-200826080-00004 18620460

[25] de Bekker-Grob EW Ryan M Gerard K. Discrete choice experiments in health economics: a review of the literature. Health Econ. (2012) 21:145–72. 10.1002/hec.1697 22223558

[26] Lancaster KJ. A new approach to consumer theory. J Polit Econ. (1966) 74:132–57. 10.1086/259131

[27] Levine DM Paz M Burke K Schnipper JL. Predictors and reasons why patients decline to participate in home hospital: a mixed methods analysis of a randomized controlled trial. J Gen Intern Med. (2022) 37:327–31. 10.1007/s11606-021-06833-2 33954888 PMC8811077

[28] Huisman-de Waal G van Achterberg T Jansen J Wanten G Schoonhoven L. “High-tech” home care: overview of professional care in patients on home parenteral nutrition and implications for nursing care. J Clin Nurs. (2011) 20:2125–34. 10.1111/j.1365-2702.2010.03682.x 21615572

[29] Welch HG Wennberg DE Welch WP. The use of Medicare home health care services. N Engl J Med. (1996) 335:324–9. 10.1056/NEJM199608013350506 8663855

[30] Wang H Zhang Y Yue S. Exploring barriers to and facilitators of the implementation of home rehabilitation care for older adults with disabilities using the Consolidated Framework for Implementation Research (CFIR). BMC Geriatr. (2023) 23:292. 10.1186/s12877-023-03976-1 37179304 PMC10183114

[31] Kadakia KT Balatbat CA Siu AL Cohen IG Wilkins CH Dzau VJ. Hospital-at-home: multistakeholder considerations for program dissemination and scale. Milbank Q. (2022) 100:673–701. 10.1111/1468-0009.12586 36148893 PMC9576240

[32] Shanghai Municipal Health Commission. Policy Interpretation of the Shanghai Hospital at Measures. (2019). Available online at: https://wsjkw.sh.gov.cn/zcjd/20191226/e8568d3c18d54015a8617206b112ca75.html (accessed January 28, 2024).

[33] Zheng X Luo Y Su B He P Guo C Tian Y. Developmental gerontology and active population aging in China. China CDC Weekly. (2023) 5:184. 37008673 10.46234/ccdcw2023.033 PMC10061737

[34] Feng Z Liu C Guan X Mor V. China's rapidly aging population creates policy challenges in shaping a viable long-term care system. Health Aff. (2012) 31:2764–73. 10.1377/hlthaff.2012.0535 23213161 PMC3711106

[35] Zhang J Xu X Yang L Wang J. Met and unmet care needs of home-living people with dementia in China: an observational study using the Camberwell Assessment of Need for the Elderly. Geriatr Gerontol Int. (2021) 21:102–7. 10.1111/ggi.14093 33238328 PMC7839676

[36] Liu X Liu Z Zheng R Li W Chen Q Cao W. Exploring the needs and experiences of palliative home care from the perspectives of patients with advanced cancer in China: a qualitative study. Support Care Cancer. (2021) 29:4949–56. 10.1007/s00520-021-06037-8 33569672

[37] World Health Organization. Integrated Care for Older People: Guidelines on Communitylevel Interventions to Manage Declines in Intrinsic Capacity. (2017). Available online at: https://www.who.int/publications/i/item/9789241550109 (accessed February 2, 2024). 29608259

[38] Nan Cheng L Zhao L Feng Xie X Wang L Ying Hu X Yang Dong X. Care willingness and demand of residents under 60 years of age in western China: a cross-sectional study. Br Med J Open. (2021) 11:e046515. 10.1136/bmjopen-2020-046515 34344676 PMC8336120

[39] Yan G Zhao L Xia L Wu J Sun L. An investigation into the needs of community health service for residents in Tianjin. J Clin Nurs Res. (2018) 2:415. 10.26689/jcnr.v2i4.415

[40] Hu R Gu B Tan Q Xiao K Li X Cao X. The effects of a transitional care program on discharge readiness, transitional care quality, health services utilization and satisfaction among Chinese kidney transplant recipients: a randomized controlled trial. Int J Nurs Stud. (2020) 110:103700. 10.1016/j.ijnurstu.2020.103700 32739670

[41] Walsh S O'Shea E Pierse T Kennelly B Keogh F Doherty E. Public preferences for home care services for people with dementia: a discrete choice experiment on personhood. Soc Sci Med. (2020) 245:112675. 10.1016/j.socscimed.2019.112675 31760321

[42] Goossens LM Utens CM Smeenk FW Donkers B van Schayck OC Rutten-van Mölken MP. Should I stay or should I go home? A latent class analysis of a discrete choice experiment on hospital-at-home. Value Health. (2014) 17:588–96. 10.1016/j.jval.2014.05.004 25128052

[43] Landers S Madigan E Leff B Rosati RJ McCann BA Hornbake R. The future of home health care: a strategic framework for optimizing value. Home Health Care Manag Pract. (2016) 28:262–78. 10.1177/1084822316666368 27746670 PMC5052697

[44] Alayli-Goebbels AF Dellaert BG Knox SA Ament AJ Lakerveld J Bot SD. Consumer preferences for health and nonhealth outcomes of health promotion: results from a discrete choice experiment. Value Health. (2013) 16:114–23. 10.1016/j.jval.2012.08.2211 23337222

[45] TJJ. Interpretation of the Main Data Results of the Seventh National Population Census of Wuhan. (2021). Available online at: https://tjj.wuhan.gov.cn/ztzl_49/pczl/202109/t20210916_1779167.shtml (accessed September 16, 2021).

[46] Kunming Municipal Bureau of Statistics. Kunming Statistical Bulletin on National Economic and Social Development. (2022). Available online at: https://www.km.gov.cn/c/2022-06-13/4413027.shtml (accessed June 13, 2022).

[47] Kunming Daily. The Main Data of the Seventh National Population Census of Kunming Was Released. (2021). Available online at: https://www.km.gov.cn/c/2021-06-07/3968656.shtml (accessed June 07, 2021).

[48] Clark MD Determann D Petrou S Moro D de Bekker-Grob EW. Discrete choice experiments in health economics: a review of the literature. Pharmacoeconomics. (2014) 32:883–902. 10.1007/s40273-014-0170-x 25005924

[49] Sarkar P Etemad A. Cardiogan: attentive generative adversarial network with dual discriminators for synthesis of ECG from PPG. In: Proceedings of the AAAI Conference on Artificial Intelligence (2021).

[50] Pourpanah F Etemad A. Exploring the landscape of ubiquitous in-home health monitoring: a comprehensive survey. arXiv preprint arXiv:230612660. (2023). 10.48550/arXiv.2306.12660

[51] Provincial Medical Insurance Bureau. Notice of Zhejiang Provincial Medical Security Bureau on Improving Home Medical Service Prices and Medical Insurance Payment Policies. (2022). Available online at: http://ybj.zj.gov.cn/art/2022/12/12/art_1229113757_2450914.html (accessed December 12, 2022).

[52] Hu B Li B Wang J Shi C. Home and community care for older people in urban China: receipt of services and sources of payment. Health Soc Care Community. (2019) 2019:12856. 10.1111/hsc.12856 31508864

[53] Song Y Anderson RA Corazzini KN Wu B. Staff characteristics and care in Chinese nursing homes: a systematic literature review. Int J Nurs Sci. (2014) 1:423–36. 10.1016/j.ijnss.2014.10.003

[54] Wang Z Xing Y Yan W Sun X Zhang X Huang S. Effects of individual, family and community factors on the willingness of institutional elder care: a cross-sectional survey of the elderly in China. Br Med J Open. (2020) 10:32478. 10.1136/bmjopen-2019-032478 32075825 PMC7044895

[55] Wang Q Abiiro GA Yang J Li P De Allegri M. Preferences for long-term care insurance in China: results from a discrete choice experiment. Soc Sci Med. (2021) 281:114104. 10.1016/j.socscimed.2021.114104 34126290

[56] Orme BK. Getting Started With Conjoint Analysis: Strategies for Product Design and Pricing Research. 2nd Edn. Madison, WI: Research Publishers (2005).

[57] Herbild L Bech M Gyrd-Hansen D. Estimating the Danish populations' preferences for pharmacogenetic testing using a discrete choice experiment. The case of treating depression. Value Health. (2009) 4:560–7. 10.1111/j.1524-4733.2008.00465.x 18980634

[58] Donnan JR Johnston KM Chibrikov E Marra CA Aubrey-Bassler K Najafzadeh M. Capturing adult patient preferences toward benefits and risks of second-line antihyperglycemic medications used in type 2 diabetes: a discrete choice experiment. Can J Diabet. (2020) 4:14. 10.1016/j.jcjd.2019.04.014 31311729

[59] Campbell D Erdem S. Including opt-out options in discrete choice experiments: issues to consider. Patient. (2018) 12:1–14. 10.1007/s40271-018-0324-6 30073482

[60] Ryan M Skåtun D. Modelling non-demanders in choice experiments. Health Econ. (2004) 4:397–402. 10.1002/hec.821 15067675

[61] Loomis JB González-Cabán A Gregory RS. Do reminders of substitutes and budget constraints influence contingent valuation estimates. Land Econ. (1994) 70:499–506. 10.2307/3146643

[62] Xue T Liu C Li Z Liu J Tang Y. Weighing patient attributes in antibiotic prescribing for upper respiratory tract infections: a discrete choice experiment on primary care physicians in Hubei Province, China. Front Public Health. (2022) 10:1008217. 10.3389/fpubh.2022.1008217 36605239 PMC9807867

[63] Walker JL Wang Y Thorhauge M Ben-Akiva M. D-efficient or deficient? A robustness analysis of stated choice experimental designs. Theory Decision. (2018) 84:215–38. 10.1007/s11238-017-9647-3

[64] Hensher DA Greene WH. The Mixed Logit model: the state of practice. Transportation. (2003) 30:133–76. 10.1023/A:1022558715350

[65] Kruk ME Johnson JC Gyakobo M Agyei-Baffour P Asabir K Kotha SR. Rural practice preferences among medical students in Ghana: a discrete choice experiment. Bull World Health Organ. (2010) 88:333–41. 10.2471/BLT.09.072892 20458371 PMC2865662

[66] Louviere JJ Flynn TN Carson RT. Discrete choice experiments are not conjoint analysis. J Choice Model. (2010) 3:57–72. 10.1016/S1755-5345(13)70014-9

[67] Gonzalez JM. A guide to measuring and interpreting attribute importance. Patient. (2019) 12:287–95. 10.1007/s40271-019-00360-3 30906968

[68] Cerwick DM Gkritza K Shaheed MS Hans ZN. A comparison of the mixed logit and latent class methods for crash severity analysis. Analyt Methods Accid Res. (2014) 3:11–27. 10.1016/j.amar.2014.09.002

[69] Greene WH Hensher DA. A latent class model for discrete choice analysis: contrasts with mixed logit. Transport Res B Methodol. (2003) 37:681–98. 10.1016/S0191-2615(02)00046-2

[70] Sarrias M Daziano RA. Individual-specific point and interval conditional estimates of latent class logit parameters. Econometrics. (2017) 2017:3065365. 10.2139/ssrn.3065365

[71] Boxall PC Adamowicz WL. Understanding heterogeneous preferences in random utility models: a latent class approach. Environ Resour Econ. (2002) 23:421–46. 10.1023/A:1021351721619

[72] Hole AR. Modelling heterogeneity in patients' preferences for the attributes of a general practitioner appointment. J Health Econ. (2008) 27:1078–94. 10.1016/j.jhealeco.2007.11.006 18179837

[73] Hole AR. Fitting mixed logit models by using maximum simulated likelihood. Stata J. (2007) 7:388–401. 10.1177/1536867X0700700306

[74] Yoo HI. lclogit2: an enhanced command to fit latent class conditional logit models. Stata J. (2020) 20:405–25. 10.1177/1536867X20931003

[75] Thomé B Dykes AK Hallberg IR. Home care with regard to definition, care recipients, content and outcome: systematic literature review. J Clin Nurs. (2003) 12:860–72. 10.1046/j.1365-2702.2003.00803.x 14632979

[76] Xiao F Cao S Xiao M Xie L Zhao Q. Patterns of home care and community support preferences among older adults with disabilities in China: a latent class analysis. BMC Geriatr. (2023) 23:117. 10.1186/s12877-023-03830-4 36869322 PMC9983178

[77] Cain CH Neuwirth E Bellows J Zuber C Green J. Patient experiences of transitioning from hospital to home: an ethnographic quality improvement project. J Hosp Med. (2012) 7:382–7. 10.1002/jhm.1918 22378714

[78] Morton RL Snelling P Webster AC Rose J Masterson R Johnson DW. Dialysis modality preference of patients with CKD and family caregivers: a discrete-choice study. Am J Kidney Dis. (2012) 60:102–11. 10.1053/j.ajkd.2011.12.030 22417786

[79] Shepperd S Gonçalves-Bradley DC Straus SE Wee B. Hospital at home: home-based end-of-life care. Cochrane Database Syst Rev. (2021) 3:CD009231. 10.1002/14651858.CD009231.pub3 33721912 PMC8092626

[80] Candy B Holman A Leurent B Davis S Jones L. Hospice care delivered at home, in nursing homes and in dedicated hospice facilities: a systematic review of quantitative and qualitative evidence. Int J Nurs Stud. (2011) 48:121–33. 10.1016/j.ijnurstu.2010.08.003 20846650

[81] Johnson CE McVey P Rhee JJ Senior H Monterosso L Williams B. General practice palliative care: patient and carer expectations, advance care plans and place of death-a systematic review. Br Med J Support Palliat Care. (2018) 2018:1549. 10.1136/bmjspcare-2018-001549 30045939

[82] Liu C Bartram T Leggat SG. Link of patient care outcome to occupational differences in response to human resource management: a cross-sectional comparative study on hospital doctors and nurses in China. Int J Environ Res Public Health. (2020) 17:124379. 10.3390/ijerph17124379 32570912 PMC7344802

[83] Osborne C Trammell M Vega M. Managing the transition from hospital to home after stroke: a patient and care partner guide to facilitate discharge planning. Arch Phys Med Rehabil. (2023) 104:161–4. 10.1016/j.apmr.2022.09.008 36181832

[84] Schofield I Knussen C Tolson D. A mixed method study to compare use and experience of hospital care and a nurse-led acute respiratory assessment service offering home care to people with an acute exacerbation of chronic obstructive pulmonary disease. Int J Nurs Stud. (2006) 43:465–76. 10.1016/j.ijnurstu.2005.07.002 16157339

[85] Niehoff KM Muscarella J Knostman M Sullivan M Gani A Lim H. Hospital at home: development of pharmacy services. Am J Health Syst Pharm. (2022) 79:1981–7. 10.1093/ajhp/zxac225 35977885

[86] Inzitari M Arnal C Burbano P Hoyos E Pérez S Bisquert M. Integrated care in a geriatric hospital at home in Barcelona. Int J Integr Care. (2022) 2022:22153. 10.5334/ijic.ICIC22153

[87] Pan F Wu H Liu C Zhang X Peng W Wei X. Effects of home telemonitoring on the control of high blood pressure: a randomised control trial in the Fangzhuang Community Health Center, Beijing. Aust J Prim Health. (2018) 24:398–403. 10.1071/PY17187 30131099

[88] Ishii S Tanabe K Ishimaru B Kitahara K. Impact of COVID-19 on long-term care service utilization of older home-dwelling adults in Japan. J Am Med Dir Assoc. (2023) 24:156–63.e23. 10.1016/j.jamda.2022.12.008 36592936 PMC9742200

[89] Haki C Deniz O. The impact of home quarantine during COVID-19 lockdown on neurological hospitalizations, in-hospital mortality, and acute ischemic stroke management in older patients without COVID-19. Clin Neurol Neurosurg. (2022) 212:107027. 10.1016/j.clineuro.2021.107027 34839154 PMC8604567

[90] Schiff R Oyston M Quinn M Walters S McEnhill P Collins M. Hospital at Home: another piece of the armoury against COVID-19. Future Healthc J. (2022) 9:90–5. 10.7861/fhj.2021-0137 35372768 PMC8966799

[91] Paulson MR Torres-Guzman RA Avila FR Maita K Garcia JP Eldaly A. 85-year-old postsurgical complex patient successfully managed remotely at the novel Mayo Clinic's Hospital at home. Case Rep Vasc Med. (2022) 2022:1439435. 10.1155/2022/1439435 35251735 PMC8896952

[92] Bentur N. Hospital at home: what is its place in the health system? Health Policy. (2001) 55:71–9. 10.1016/S0168-8510(00)00114-7 11137189

[93] Arai H Ouchi Y Toba K Endo T Shimokado K Tsubota K. Japan as the front-runner of super-aged societies: perspectives from medicine and medical care in Japan. Geriatr Gerontol Int. (2015) 15:673–87. 10.1111/ggi.12450 25656311

[94] Braet A Weltens C Vleugels A. Effectiveness of discharge interventions from hospital to home to reduce readmissions: a systematic review. JBI Libr Syst Rev. (2012) 10:1–13. 10.11124/jbisrir-2012-310 27820399

[95] Jones J Wilson A Parker H Wynn A Jagger C Spiers N. Economic evaluation of hospital at home versus hospital care: cost minimisation analysis of data from randomised controlled trial. Br Med J. (1999) 319:1547–50. 10.1136/bmj.319.7224.1547 10591720 PMC28300

[96] Sun Y Iwagami M Komiyama J Sugiyama T Inokuchi R Sakata N. Association between types of home healthcare and emergency house calls, hospitalization, and end-of-life care in Japan. J Am Geriatr Soc. (2023) 71:1795–805. 10.1111/jgs.18268 36789967

[97] Aggarwal A Choudhury A Fearnhead NS Kearns P Kirby A Lawler M. The future of cancer care in the UK-time for a radical and sustainable National Cancer Plan. Lancet Oncol. (2023). 10.1016/S1470-2045(23)00511-9 37977167

[98] McKnight R. Home care reimbursement, long-term care utilization, and health outcomes. Health Care Deliv Fin. (2004) 2004:w10414. 10.3386/w10414

[99] Norgall T. Fit and independent in the aging population using technology. From concept to reality? Bundesgesundheitsblatt Gesundheitsforschung Gesundheitsschutz. (2009) 3:297–305. 10.1007/s00103-009-0789-5 19263030

[100] Rajput ZA Mbugua S Amadi D Chepngeno V Saleem JJ Anokwa Y. Evaluation of an Android-based mHealth system for population surveillance in developing countries. J Am Med Informat Assoc. (2012) 4:655–9. 10.1136/amiajnl-2011-000476 22366295 PMC3384107

[101] Saenger P Federman AD DeCherrie LV Lubetsky S Catalan E Leff B. Choosing inpatient vs. home treatment: why patients accept or decline hospital at home. J Am Geriatr Soc. (2020) 68:1579–83. 10.1111/jgs.16486 32374438

