# Supplementary material for: Preference of primary care patients for home-based healthcare and support services: a discrete choice experiment in China
Source: Front Public Health. 2024 Apr 18;12:1324776. doi: 10.3389/fpubh.2024.1324776 (PMC11063295; doi:10.3389/fpubh.2024.1324776)
Supplement: Supplementary file 1 [file Data_Sheet_1.pdf]

Supplementary Material

1 APPENDIX A : Policy document review

63 policy documents related to home-based healthcare and support services were identified from the official websites of the National Health Commission (NHC) of China, the National Health Insurance Bureau of China, the provincial and municipal health administrative departments, and various levels of governments from January 1984 to June 2023 (Table A1).

Texts in the 63 policy documents were coded word by word and sentence by sentence using Nvivo11 software (Table A2). The codes were compared, refined, and categeorized into eight themes (Table A3). Five of the eight themes were deemed important for inclusion in the DCE design considering their relevance to the patients (Table A4).

Supplementary material is not typeset so please ensure that all information is clearly presented, the appropriate caption is included in the file and not in the manuscript, and that the style conforms to the rest of the article.

Table A1. Policy documents identified

No	**Title**	**Date**	**Governmental agency**	**Application**
1	National Nursing Development Plan (2021-2025)	April 29, 2022	NHC	Nationwide
2	Notice on Strengthening Home-based Medical Services for the Elderly	Dec 17, 2020	Office of the State Administration of Traditional Chinese Medicine, NHC	Nationwide
3	Guiding Opinions on Establishing and Improving the Health Service	Oct 28, 2019	NHC, National Development and Reform Commission, Ministry of Education, Ministry of Civil Affairs,	Nationwide

	System for the Elderly		Ministry of Finance, Ministry of Human Resources and Social Security, National Medical Insurance Bureau, State Administration of Traditional Chinese Medicine	
4	Guidelines on Further Promoting the Joint Development of Medical and Nursing Care	July 18, 2022	NHC, National Development and Reform Commission, Ministry of Education, Ministry of Civil Affairs, Ministry of Finance, Ministry of Human Resources and Social Security, Ministry of Natural Resources, Ministry of Housing and Urban-Rural Development, Ministry of Emergency Responses, General Administration of Market Regulation, State Medical Insurance Bureau	
5	Notice on Strengthening Comprehensive Health Services for the Elderly	Dec 31, 2021	National Office on Aging, NHC, National Bureau of Traditional Chinese Medicine	Nationwide
6	Notice on issuing Guidelines on Promoting Reform and Development of Nursing Service Industry	June 21, 2018	NHC, National Development and Reform Commission, Ministry of Education, Ministry of Civil Affairs, Ministry of Finance, Ministry of Human Resources and Social Security, State Administration for	Nationwide

			Market Supervision and Regulation of China, Banking and Insurance Regulatory Commission, State Administration of Traditional Chinese Medicine, Chinese Federation of People With Disability, Logistics Security Department of Central Military Commission	
7	Notice on Issuing Opinions on Accelerating the Development of Rehabilitation Medical Work	June 8, 2021	NHC, National Development and Reform Commission, Ministry of Education, Ministry of Civil Affairs, Ministry of Finance, National Medical Insurance Bureau, National Traditional Chinese Medicine Bureau, Chinese Federation of People with disability	
8	Notice on Printing and distributing Guidelines for Signing Cooperative Services between Medical/Health Institutions and Aged Care Institutions (Trial)	Dec 11, 2020	Office of the State Administration of Traditional Chinese Medicine, General Office of the Ministry of Civil Affairs, NHC	Nationwide
9	Temporary Work Regulations on	December 15, 1984	Ministry of Health	Nationwide

	Hospital at Home			
10	Notice on Carrying out Pilot work on Medical and Nursing Services for the Elderly	Nov 15, 2021	General Office of the NHC	Beijing, Shanxi, etc
11	Implementation Plan on Strengthening Home-based Medical Services for the Elderly	Jan 14, 2021	Hebei Provincial Health Commission, Hebei Provincial Administration of Traditional Chinese Medicine	Hebei Province
12	Implementation Plan for Strengthening Home-based Medical Services for the Elderly in Heilongjiang Province	May 12, 2021	Heilongjiang Provincial Administration of Traditional Chinese Medicine, Heilongjiang Provincial Health Commission	Heilongjiang Province
13	Implementation Plan of Hospital at Home Delivered by Primary care Institutions	Nov 22, 2017	Daqing Human Resources and Social Security Bureau, Daqing Oilfield Social Insurance Center, Daqing Health and Family Planning Commission	Daqing City, Heilongjiang Province
14	Implementation Plan for Hospital at Home Services	Nov 18, 2021	Jintan District Health Bureau, Changzhou	Changzhou City, Jiangsu Province
15	Regulations on the Administration of Hospital at Home (Revised)	July 15, 2016	Suzhou Municipal Health and Family Planning Commission	Suzhou, Jiangsu Province

16	Notice on Improving Home-based Medical Service Price and Insurance Payment Policy	Dec 2, 2022	Zhejiang Health Security Bureau	Zhejiang Province
17	Conditions and Procedures for Establishing "Home Bed"	Nov 24, 2014	Hangzhou Human Resources and Social Security Bureau, Hangzhou Health and Family Planning Commission	Hangzhou, Zhejiang Province
18	Code of Hospital at Home Established by Community Health Institutions (Trial)	June 19, 2017	Health and Family Planning Commission of Shaoxing City	Shaoxing, Zhejiang Province
19	Implementation measures of Hospital at Home Management (Trial)	Dec 13, 2018	Wenzhou Health and Family Planning Commission, Wenzhou Human Resources and Social Security Bureau, Wenzhou Finance Bureau, Wenzhou Development and Reform Commission	Wenzhou, Zhejiang Province
20	Implementation Plan on Building Family Nursing Beds for the Aged	October 10, 2022	Ningbo Finance Bureau, Ningbo Civil Affairs Bureau	Ningbo, Zhejiang province
21	Hospital at Home Standards (Trial)	Dec 14, 2016	Hefei Human Resources and Social Security Bureau, Hefei Municipal Health and Family Planning Commission	Hefei, Anhui province

22	Service Standards of Home Beds (Trial)	September 2, 2019	Tunxi District Health Commission of Huangshan City	Huangshan City, Anhui Province
23	Implementation Plan for Pilot Work of Residential Nursing Beds	May 6, 2022	Suzhou Municipal Bureau of Finance	Suzhou, Anhui Province
24	Implementation Plan for the pilot work of home aged care beds	May 31, 2022	Suzhou Civil Affairs Bureau	Suzhou, Anhui Province
25	Notice on Regulating the Management and Services of Hospital at Home	April 30, 2021	Fujian Medical Security Bureau, Fujian Provincial Health Commission	Fujian Province
26	Notice on Issues Related to Standardizing Charges for Hospital at Home	Dec 28, 2020	Health and Health Commission of Fujian Province, Medical Security Bureau	Fujian Province
27	Notice on Regulating the Management and Services of Hospital at Homes (Draft for Comment)	July 1, 2021	Xiamen Municipal Health Commission	Xiamen, Fujian Province
28	Notice on Regulating the Management and Services of Hospital at Home	June 30, 2021	Longyan Medical Security Bureau, Longyan Health Commission	Longyan City, Fujian province

29	Measures for the Construction and Services of Family Aged Care Beds	Nov 9, 2021	Fuzhou Civil Affairs Bureau, Fuzhou Finance Bureau	Fuzhou, Fujian Province
30	Pilot Work Plan for Family Aged care Beds	June 15, 2021	Nanchang Civil Affairs Bureau	Nanchang City, Jiangxi Province
31	Pilot Work Plan of Home-based Medical Services for the Elderly	June 25, 2021	Shandong Provincial Health Commission	Shandong Province
32	Implementation Plan of Home-based Medical Services for the Elderly	Aug 6, 2021	Qingdao Health Commission	Qingdao, Shandong Province
33	Measures for the Administration of Home Beds Established by Urban Community Health Services (Trial)	November 9, 2009	Hunan Provincial Health Commission	Hunan Province
34	Measures for the Implementation of Basic Medical Insurance	September 11, 2017	Jingmen People's Government	Jingmen, Hubei Province
35	Work Plan for Pilot Beds for Elderly Families	April 22, 2021	Wuhan Civil Affairs Bureau, Wuhan Finance Bureau	Wuhan, Hubei Province
36	Management Standards for Hospital at Home in Community Health Services (Trial)	October 16, 2013	Guangzhou Municipal Health and Family Planning Commission	Guangzhou, Guangdong Province

37	Guidelines on the Administration of Hospital at Home (Trial)	Dec 15, 2014	Guangdong Provincial Health Commission	Guangdong Province
38	Guidelines on Hospital at Home	Aug 12, 2016	Guangzhou Medical Insurance Bureau, Guangzhou Municipal Health Commission	Guangdong Province
39	Guidelines on Home-based Medical and Health Services for the Elderly	May 6, 2020	Guangdong Provincial Health Commission, Guangdong Provincial Civil Affairs Department	Guangdong Province
40	Measures for the Administration of Hospital at Home (Trial)	Feb 9, 2021	Shenzhen Medical Insurance Bureau, Shenzhen Municipal Health Commission	Shenzhen, Guangdong Province
41	Local Standards for Smart Hospital at Home (Standard DB4403/T 42-2020)	March 17, 2021	Shenzhen Municipal Health Commission	Shenzhen, Guangdong Province
42	Home Bed Management Service Standards (Trial)	Nov 22, 2019	Sichuan Provincial Health Commission	Sichuan Province
43	"Hospital at Home" conditions and procedures	Oct 23, 2015	Qianwei County Health Bureau, Leshan City, Sichuan province	Qianwei County, Leshan City, Sichuan province
44	Work Plan of Medical Care Services for the Elderly at Home	May 30, 2022	Guizhou Provincial Health Commission	Guizhou Province

45	Implementation Plan for Strengthening Home-based Medical Services for the Elderly	Jan 24, 2021	Health Commission of Inner Mongolia Autonomous Region	Inner Mongolia Autonomous Region
46	Interim Measures for Basic Medical Insurance for Urban and Rural Residents (Abstract)	Nov 3, 2020	Medical Security Bureau of Guangxi Zhuang autonomous region	Guangxi Zhuang Autonomous Region
47	Interim Measures for Basic Medical Insurance for Employees (Abstract)	July 10, 2020	Beihai City Medical Security Bureau	Beihai City, Guangxi Zhuang autonomous region
48	Measures for the Administration of Hospital at Home under Basic Medical Insurance for Urban Employees	May 3, 2013	Beihai City Human Resources and Social Security Bureau	Beihai City, Guangxi Zhuang autonomous region
49	Hospital at Home Treatment Qualification Requirements	May 1, 2019	Beihai City Human Resources and Social Security Bureau	Beihai City, Guangxi Zhuang autonomous region
50	Guiding Opinions on Pilot work of Hospital at Home	March 8, 2121	Health Commission of Ningxia Hui Autonomous Region	Ningxia Hui Autonomous Region
51	Implementation Plan for Further Deepening the Reform of Basic Medical	Oct 19, 2018	Zhongwei Municipal People's Government Office	Zhongwei City, Ningxia Hui autonomous region

	Insurance Payment Methods			
52	Notice on Pilot Work for Home-based Aged Care	June 3, 2009	Bayingoleng Prefecture Civil Affairs Bureau	Bayingoleng Mongolian Autonomous Prefecture, Xinjiang Uygur Autonomous Region
53	Rules for the Implementation of Preferential Treatment for the Elderly in Medical Institutions (Trial Implementation)	December 23, 2008	Beijing Municipal Commission of Health and Family Planning, Office of Beijing Municipal Working Committee on Aging, Beijing Municipal Health Bureau	Beijing
54	Guidelines on Community Health Services to Support home-based Care Services	Dec 7, 2015	Beijing Municipal Commission of Health and Family Planning	Beijing
55	Notice on Carrying out the Management Project for the Elderly with Disability and Mental Disorders	May 11, 2022	Beijing Municipal Health Commission	Beijing
56	Notice on Developing and Regulating Internet Home Care Services	Dec 26, 2018	Beijing Municipal Health Commission, Beijing Municipal Administration of Market Supervision, Beijing Municipal Medical Security Bureau	Beijing

57	Notice on Further Improving Medical Services	March 15, 2017	Beijing Municipal Commission of Health and Family Planning	Beijing
58	Notice on Improving the Quality of Family Doctors Contract Services	Sept 26, 2017	Beijing Municipal Commission of Health and Family Planning	Beijing
59	Measures for the Administration of Construction of Home Care Beds for the Elderly (Trial)	March 26, 2021	Beijing Social Work Committee, Beijing Municipal Civil Affairs Bureau, Beijing Municipal Finance Bureau, Beijing Municipal Health Commission, Beijing Municipal Medical Security Bureau, Beijing Federation of People with Disability	Beijing
60	Work Plan for Improving the Quality and Scale of Family Doctor Contract Services	June 30, 2021	Beijing Municipal Health Commission, Beijing Municipal Commission of Development and Reform, Beijing Municipal Commission of Science and Technology, Beijing Municipal Bureau of Economy and Information Technology, Beijing Municipal Bureau of Civil Affairs, Beijing Municipal Bureau of Finance, Beijing Municipal Bureau of Human Resources and Social Security, Beijing Municipal	Beijing

			Commission of Planning and Natural Resources, Beijing Municipal Medical Security Bureau, Beijing Municipal Administration of Traditional Chinese Medicine	
61	Internet Home Care Service Project Directory (2022 edition)	September 30, 2022	Beijing Municipal Health Commission	Beijing
62	Standards for Hospital at Home (DB31/T487-2010)	September 20, 2010	Shanghai Health Bureau	Shanghai
63	Measures on Hospital at Home	Dec 10, 2019	Shanghai Municipal Health Commission	Shanghai

表 A1 中国 63 份家庭病床服务相关政策文本来源

序号	**地区**	**文件名**	**发文时间**	**发文机构**
1	全国	《全国护理事业发展规划（2021—2025 年）》	2022 年 4 月 29 日	国家卫生健康委
2		《关于加强老年人居家医疗服务工作的通知》	2020 年 12 月 17 日	国家卫生健康委办公厅 国家中医药管理局办公室
3		《关于建立完善老年健康服务体系的指导意见》	2019 年 10 月 28 日	国家卫生健康委 国家发展改革委 教育部 民政部 财政部 人力资

				源社会保障部 国家医保局　国家中医药局
4		《关于进一步推进医养结合发展的指导意见》	2022 年 7 月 18 日	国家卫生健康委 国家发展改革委 教育部 民政部 财政部 人力资源社会保障部 自然资源部 住房城乡建设部 应急部　市场监管总局 国家医保局
5		《关于全面加强老年健康服务工作的通知》	2021 年 12 月 31 日	国家卫生健康委 全国老龄办 国家中医药局
6		《关于印发促进护理服务业改革与发展指导意见的通知》	2018 年 6 月 21 日	国家卫生健康委员会 国家发展和改革委员会 教育部<br>民政部 财政部 人力资源和社会保障部 国家市场监督管理总局 中国银行保险监督管理委员会 国家中医药管理局 中国残疾人联合会 中央军委后勤保障部
7		《关于印发加快推进康复医疗工作发展意见的通知》	2021 年 6 月 8 日	国家卫生健康委　国家发展改革委 教育部 民政部 财政部　国家医保局 国家中医药局　中国残联
8		《关于印发医疗卫生机构与养老服务机构签约合作服务指南（试行）的通知》	2020 年 12 月 11 日	国家卫生健康委办公厅 民政部办公厅 国家中医药管理局办公室
9		《家庭病床暂行工作条例》	1984 年 12 月 15 日	国家原卫生部

10	北京市、山西省等	《关于开展老年医疗护理服务试点工作的通知》	2021年11月15日	国家卫生健康委办公厅
11	河北省	《河北省关于加强老年人居家医疗服务工作实施方案》	2021年1月14日	河北省卫生健康委 河北省中医药管理局
12	黑龙江省	《黑龙江省加强老年人居家医疗服务工作实施方案》	2021年5月12日	黑龙江省卫生健康委员会 黑龙江省中医药管理局
13	黑龙江省大庆市	《大庆市基层医疗卫生机构开展家庭病床服务工作实施方案》	2017年11月22日	大庆市卫生和计划生育委员会 大庆市人力资源和社会保障局、大庆油田社会保险中心
14	江苏省常州市	《常州市金坛区家庭病床预约上门服务实施方案》	2021年11月18日	常州市金坛区卫生健康局
15	江苏省苏州市	苏州市家庭病床服务管理规定（修订）	2016年7月15日	苏州市卫生和计划生育委员会
16	浙江省	《关于完善居家医疗服务价格和医保支付政策的通知》	2022年12月2日	浙江省医疗保障局
17	浙江省杭州市	《浙江省杭州市申请“家庭病床”条件及流程》	2014年11月24日	杭州市卫生和计划生育委员会 杭州市人力资源和社会保障局
18	浙江省绍兴市	《绍兴市社区卫生服务机构家庭病床服务规范（试行）》	2017年6月19日	绍兴市原卫生计生委

19	浙江省温州市	《温州市家庭病床管理实施办法（试行）》	2018 年 12 月 13 日	温州市卫生和计划生育委员会 温州市人力资源社会保障局 温州市财政局 温州市发改委
20	浙江省宁波市	《关于开展家庭养老照护床位建设的实施方案》	2022 年 10 月 10 日	宁波市财政局 宁波市民政局
21	安徽省合肥市	《合肥市家庭病床服务规范（试行）》	2016 年 12 月 14 日	合肥市卫生和计划生育委员会 合肥市人力资源与社会保障局
22	安徽省黄山市	《黄山市屯溪区家庭病床服务规范（试行）》	2019 年 9 月 2 日	黄山市屯溪区卫生健康委员会
23	安徽省宿州市	《宿州市家庭养老床位试点工作实施方案》	2022 年 5 月 6 日	宿州市财政局
24	安徽省宿州市	《埇桥区家庭养老床位试点工作实施方案》	2022 年 5 月 31 日	宿州市民政局
25	福建省	《关于规范我省家庭病床管理和服务的通知》	2021 年 4 月 30 日	福建省卫生健康委员会 福建省医疗保障局
26	福建省	《关于规范家庭病床服务收费有关问题的通知》	2020 年 12 月 28 日	福建省医疗保障局 福建省卫生健康委员会
27	福建省厦门市	《关于规范厦门市家庭病床管理和服务的通知（征求意见稿）》	2021 年 7 月 1 日	厦门市卫生健康委员会
28	福建省龙岩市	《关于规范我市家庭病床管理和服务的通知》	2021 年 6 月 30 日	龙岩市卫生健康委员会 龙岩市医疗保障局

29	福建省福州市	《福州市家庭养老床位建设和服务实施办法》	2021 年 11 月 9 日	福州市民政局 福州市财政局
30	江西省南昌市	《南昌市家庭养老照护床位试点工作方案》	2021 年 6 月 15 日	南昌市民政局
31	山东省	《山东省老年人居家医疗服务试点工作方案》	2021 年 6 月 25 日	山东省卫生健康委员会
32	山东省青岛市	《青岛市老年人居家医疗服务实施方案》	2021 年 8 月 6 日	青岛市卫生健康委员会
33	湖南省	《湖南省城市社区卫生服务家庭病床管理办法（试行）》	2009 年 11 月 9 日	湖南省卫生健康委员会
34	湖北省	《荆门市基本医疗保险实施办法》	2017 年 9 月 11 日	荆门市人民政府
35	湖北省武汉市	《武汉市家庭养老床位试点工作方案》	2021 年 4 月 22 日	武汉市民政局 武汉市财政局
36	广东省广州市	《广州市社区卫生家庭病床服务管理规范 （试行）》	2013 年 10 月 16 日	广州市卫生和计划生育委员会
37	广东省	《广东省开展家庭病床服务管理指引（试行）》	2014 年 12 月 15 日	广东省卫生健康委员会
38	广东省	《广东省开展家庭病床服务工作指引》	2016 年 8 月 12 日	广州市卫生健康委员会 广州市医保局

39	广东省	《广东省老年人居家医疗健康服务工作指引》	2020 年 5 月 6 日	广东省卫生健康委 广东省民政厅
40	广东省深圳市	《深圳市家庭病床管理办法（试行）》	2021 年 2 月 9 日	深圳市卫生健康委员会 深圳市医保局
41	广东省深圳市	《深圳市地方标准智慧家庭病床服务规范 DB4403/T 42—2020 》	2021 年 3 月 17 日	深圳市卫生健康委员会
42	四川省	《四川省家庭病床管理服务规范（试行）》	2019 年 11 月 22 日	四川省卫生健康委员会
43	四川省乐山市犍为县	四川省乐山市犍为县申请“家庭病床”条件及流程	2015 年 10 月 23 日	四川省乐山市犍为县卫生健康局
44	贵州省	《贵州省居家老年人医养服务工作方案》	2022 年 5 月 30 日	贵州省卫生健康委员会
45	内蒙古自治区	《内蒙古自治区加强老年人居家医疗服务工作实施方案》	2021 年 1 月 24 日	内蒙古自治区卫生健康委
46	广西壮族自治区	《广西城乡居民基本医疗保险暂行办法（摘要）》	2020 年 11 月 3 日	广西壮族自治区医疗保障局
47	广西壮族自治区北海市	《广西北部湾经济区职工基本医疗保险暂行办法（摘要）》	2020 年 7 月 10 日	北海市医疗保障局

48	广西壮族自治区北海市	《北海市城镇职工基本医疗保险家庭病床管理办法》	2013年5月3日	北海市人力资源和社会保障局
49	广西壮族自治区北海市	《北海市家庭病床待遇资格申请》	2019年5月1日	北海市人力资源和社会保障局
50	宁夏回族自治区	《关于在全区开展家庭病床试点工作的指导意见》	2121年3月8日	宁夏回族自治区卫生健康委员会
51	宁夏回族自治区中卫市	《中卫市进一步深化基本医疗保险支付方式改革实施方案》	2018年10月19日	中卫市人民政府办公室
52	新疆维吾尔自治区巴音郭楞蒙古自治州	《关于开展居家养老试点工作的通知》	2009年6月3日	巴音郭楞蒙古自治州民政局
53	北京市	《医疗机构落实老年人优待工作的实施细则（试行）》	2008年12月23日	北京市卫生局 北京市老龄工作委员会办公室 北京市卫生和计划生育委员会
54	北京市	《北京市社区卫生服务机构支持居家养老服务的指导意见》	2015年12月7日	北京市卫生和计划生育委员会
55	北京市	《北京市卫生健康委员会关于开展2022年失能失智老年人管理项目的通知》	2022年5月11日	北京市卫生健康委员会

56	北京市	《关于发展和规范互联网居家护理服务的通知》	2018 年 12 月 26 日	北京市卫生健康委员会 北京市市场监督管理局 北京市医疗保障局
57	北京市	《关于进一步改善医疗服务的通知》	2017 年 3 月 15 日	北京市卫生和计划生育委员会
58	北京市	《关于提升北京市家庭医生签约服务质量有关工作的通知》	2017 年 9 月 26 日	北京市卫生和计划生育委员会
59	北京市	《北京市养老家庭照护床位建设管理办法（试行）》	2021 年 3 月 26 日	中共北京市委社会工作委员会 北京市民政局 北京市财政局 北京市卫生健康委员会 北京市医疗保障局 北京市残疾人联合会
60	北京市	《北京市提升家庭医生签约服务质量与规模工作方案》	2021 年 6 月 30 日	北京市卫生健康委员会 北京市发展和改革委员会 北京市科学技术委员会 北京市经济和信息化局 北京市民政局 北京市财政局 北京市人力资源和社会保障局 北京市规划和自然资源委员会 北京市医疗保障局 北京市中医管理局
61	北京市	《北京市互联网居家护理服务项目目录（2022 版）》	2022 年 9 月 30 日	北京市卫生健康委员会
62	上海市	《上海市家庭病床服务规范（DB31/T487-2010）》	2010 年 9 月 20 日	上海市卫生局
63	上海市	《上海市家庭病床服务办法》	2019 年 12 月 10 日	上海市卫生健康委员会

Table A2. Coding chart for the 63 policy documents

Coding	First level node	Codin g	Second level nodes	Examples of specific coding
**1**	**Target population**	**1-1**	**Older people with disability**	Local people aged 75 years or older who meet infrastructure requirements for home nursing care, the family voluntarily participate in the pilot project and sign a service agreement with the service provider.
		**1-2**	**Chronic conditions**	Patients with chronic conditions that require long-term treatment, including those who are bedridden, have advanced cancer, in a vegetative state, suffer from hemiplegia with bedsore infections, urinary retention, dysphagia (requiring regular dressing changes, regular replacement of urinary tubes and stomach tubes), and those who need palliative care and live with dementia.
		**1-3**	**Post-discharge care**	Patients who still need to continue their rehabilitation during the recovery period after discharge from hospital.
			**...**	
**2**	Health institutions and professional	**2-1**	**Government departments**	The Civil Affairs Bureau is the guiding and coordinating authority for the pilot work of home-based care for the elderly. It is responsible for guiding the pilot counties to formulate relevant regulations, organizing inspection and training, and summarizing and exchanging experience. ...

		**2-2**	**Medical institution**	Medical institutions that have registered and obtained the practice license for medical interventions, including diagnosis and treatment capacities delivered at home. Primary and secondary health institutions are prioritized for home-base health and care support services.
		**2-3**	**Professional**	Physicians should have the competency consistent with the home-based medical services requirements and have at least 3 years of independent clinical work experience.
**3**	**Mode of service**	**3-1**	**Home visit**	Medical institutions provide home medical services through Hospital at Home, home visits, and contract with family doctors.
		**3-2**	**"Internet + Home Bed"**	Medical institutions extend medical services to patient’s home through medial alliance, "Internet + medical care", and telemedicine. In addition, "Internet + nursing" services can be incorporated.
**4**	**Scope of services**	**4-1**	**Health monitoring and management**	1. Establish health recodes. 2. Preventive health care, including health consultation, medication reminder, nutrition advice, etc. 3. Routine health monitoring, including body temperature, weight, blood pressure, respiration, heart rate, blood sugar, etc.
		**4-2**	**Nursing Services**	1. Cleaning, including washing, hair dressing, shaving, bathing assistance, etc.; 2. Feeding, including feeding food (water), etc.; 3. Hygiene, including care for urination, defecation, etc.; 4.

				Body movement, including assistance with turning over, back patting, bedsore prevention, etc.; 5. Training and guidance for family caregivers for personal care.
		**4-3**	**Chinese Medicine Services**	Syndrome differentiation and corresponding modality for interventions, including health education.
			**...**	
**5**	**Service fee**	**5-1**	**Fee schedule**	1. Fee for services schedule for medical and nursing services items delivered at home, such as the "Hunan Medical Service charging standard". 2. Transportation and associated human resources costs paid out of pocket in line with the market price or an agreed price.
		**5-2**	**Medical insurance reimbursement**	Relevant health insurance programs reimburse certain percentage of charges based on the eligible items covered by the fee for service schedule in line with the local policy.
		**5-3**	**Method of settlement**	Insurance settlement for the insured patients who receive post-discharge treatment at home for stroke, advanced cancer, or fracture traction required fee occurs every 90 days.
**6**	**Subsidy policy**	**6-1**	**Funding for provider**	Fee for service reimbursement is replace by a capped budget for nursing beds with a large scale and those last for more than 6 months. For example, those that are qualified are given 20,000 yuan per 10

				nursing beds by the local government.
		**6-2**	**Funding for consumer**	Governmental subsidies for aged care services can be used to pay for home-based nursing care.
**7**	**Bed Configuration**	**7-1**	**Specific configuration**	Transport vehicle with standardized requirements, mobile computer device (Internet connection required), medical packages (syringe, medicine, disinfectant, thermometer, cotton and others), stethoscope, blood pressure meter, blood glucose meter, electrocardiogram machine, blood oxygen saturation detector, electroacupuncture and electromagnetic therapeutic apparatus, air mattress, etc..
			**...**	
**8**	**Management Regulations**	**8-1**	**Home bed set up**	Eligible individuals can choose any eligible service institutions to set up home nursing beds.
		**8-2 -**	**Bed inspection**	The physical in charge carries out door-to-door bed inspections or virtually depending on the needs of the patient. Remote bed inspection can be conducted when relevant infrastructure and equipment are available.
			**...**	

表 A2 我国家庭病床服务相关政策编码

编码	一级节点	编码	二级节点	具体编码示例
**1**	**服务对象**	**1-1**	**慢性病、失能老年人**	具有本市户籍,75 周岁以上的失能老年人,具备家庭养老照护基础,老年人及其家属自愿参加试点服务,并与相关服务机构签订服务协议。
		**1-2**	**慢性病患者**	慢性疾病需长期治疗的病人。包括：长期卧床病人，晚期肿瘤、植物状态、偏瘫患者合并褥疮感染、尿潴留、吞咽困难（需定期换药、定期更换尿管、胃管）病人；临终关怀病人，晚期肿瘤、植物状态、老年期痴呆症等。
		**1-3**	**出院后患者**	出院后恢复期仍需继续康复的患者。
			**……**	
**2**	**服务主体**	**2-1**	**政府部门**	州民政局是居家养老试点工作的指导协调机关，负责指导各试点县制定相关规定，组织考察培训，总结交流经验。……
		**2-2**	**医疗机构**	已执业登记取得《医疗机构执业许可证》，具有与所开展居家医疗服务相应的诊疗科目并已具备家庭病床、巡诊等服务方式的医疗机构，重点是二级及以下医院、基层医疗卫生机构等。
		**2-3**	**医务人员**	医师应当具备与所提供居家医疗服务相符合的执业类别和执业范围，同时至少具备 3 年以上独立临床工作经验的执业医师。
**3**	**服务方式**	**3-1**	**上门服务**	医疗机构可以通过家庭病床、上门巡诊、家庭医生签约等方式提供居家医疗服务。
		**3-2**	**“互联网+家庭病床”**	（医疗机构）可通过医联体、“互联网+医疗健康”、远程医疗等将医疗机构内医疗服务延伸至居家，亦可结合全省“互联网+护理服务”试点工作同步探索、相互衔接，不断丰富“互联网+医疗健康”服务内容。

**4**	**服务内容**	**4-1**	**健康管理服务**	1.建立健康档案。2.预防保健，包括健康咨询、用药提醒、营养指导等。3.常规生理指数监测，包括体温、体重、血压、呼吸、心率、血糖等。
		**4-2**	**护理服务**	1.清洁护理，包括洗漱、剪发剃须、助浴等；2.进食护理，包括喂饭（水）等；3.排泄护理，包括排尿护理、排便护理、排气护理等；4.协助和指导翻身、拍背、褥疮预防等；5.为家庭照护者提供护理技能提升培训和指导等。
		**4-3**	**中医服务**	中医服务包括中医辨证论治、中医技术、健康指导等。
			**……**	
**5**	**服务费用**	**5-1**	**收费标准**	1.建立家庭病床和提供家庭病床医疗服务项目收费，要严格执行省物价局、省卫生厅下发的《湖南省医疗服务收费标准》。2.医护人员的交通费由家庭病床患者承担，参照市场价或协议价执行。
		**5-2**	**医保报销**	纳入当地医保支付范围后，符合医保条件的患者保内费用由医保经办部门按标准支付。
		**5-3**	**结算方式**	中风瘫痪康复期、恶性肿瘤晚期或骨折牵引需卧床治疗的参保患者，可申请设立家庭病床，所需费用按 90 天为一个结算周期。
**6**	**补助政策**	**6-1**	**补助机构**	对规模化、品牌化经营的家庭养老床位实行“以奖代补”。连续提供 6 个月以上签约服务的家庭养老床位，经评估合格的，每设置 10 张给予服务提供机构 20000 元的一次性奖励。奖励资金由各区负担，市级财政给予一定补助。
		**6-2**	**补助患者**	特殊困难老年人养老服务补贴可用于支付家庭养老床位照护服务费。
**7**	**病床配置**	**7-1**	**具体配置**	出诊车辆（建议使用统一配置、标识的交通工具）、手提电脑（须联网）、出诊箱（内置有针剂、药品、消毒液、纱块、棉 垫、棉枝、体温计）、听诊器、血压计、血糖仪、心电图机、血 氧饱和度检测仪、中频治疗仪、神灯、气垫床等，可根据实际适当增加或减少。

**8**	**管理规章**	**8-1**	**建床**	符合条件的老年人可根据民政部门公布的试点机构名单自主选择适合的服务机构，并向机构提出设立家庭养老床位申请。……
		**8-2**	**查床**	责任医生应根据服务对象的病情需要开展上门查床或远程查床，远程查床可通过相关设施设备 以远程诊疗、远程健康指导等方式开展。
			**……**	

Table A3. Attributes, options, and sources used in the DCE design

Attributes	Levels	Policy Sources
Scope of services	0=Health monitoring and management	Measures for the Management of Hospital at Home in Urban Community Health Services in Hunan Province", "Guidelines for the Development of Hospital at Home in Guangdong Province", "Measures for Hospital at Home in Shanghai", etc.
	1=Diagnosis and treatment	
	2=Care support services	
	3=Mental health services	
Professional	0=Doctor	Implementation Plan for Strengthening Home Medical Services for the Elderly in Hebei Province", " Hospital at Home in Nanchang, Jiangxi Province", "Pilot Work Plan for Home Medical Services for the Elderly in Shandong Province", etc.
	1=Nurse	
	2=Others	
Institutions	0=Hospitals	Wuhan Pilot Work Plan for Family Elderly Beds", "Guangzhou Community Hospital at Home Management Specification", "Notice of the Health and Health Commission of Inner Mongolia Autonomous Region on the Issuance of the Implementation Plan for Strengthening Home Medical Services for the Elderly in Inner Mongolia Autonomous Region", etc.
	1=Primary care institutions	
	2=Other institutions	

Insurance reimbursement rate	0=85%<br><br>1=80%<br><br>2=75%	Medical Insurance Treatment in Jingmen City, Hubei Province", "Heilongjiang Daqing Primary Health Care Institutions to Carry out the Implementation Plan of Hospital at Home Payment", etc.
Visiting fee	0=50 Yuan per visit<br><br>1=65 Yuan per visit<br><br>2=80 Yuan per visit	Implementation Plan for Strengthening Home Medical Services for the Elderly in Hebei Province", "Family Elderly Care Beds in Nanchang, Jiangxi Province", "Pilot Work Plan for Home Medical Services for the Elderly in Shandong Province", etc.

2 APPENDIX B: The code used in the NGene.

```
Design

; alts = sitA, sitB

; rows = 36

; block = 3

; orth = ood

; model:

U(sitA) = b1 +

b2[0|0|0].effects*A[0,1,2,3] +

b3[0|0].effects*B[0,1,2] +

b4[0|0].effects*C[0,1,2] +

b5[0|0].effects*D[0,1,2] +

b6[0|0].effects*E[0,1,2]

/

U(sitB) = b2*A + b3*B + b4*C + b5*D + b6*E$
```

3 APPENDIX C: The code used in Stata

```
*mixlogit model

ssc install mixlogit

 mixlogit choice, group(obsid) id(personid) rand(sev1 sev2 sev3 fac1 fac2 sta1 sta2 expense ratio) nrep(500)

*AIC and BIC

local ll = e(ll)

local df_m = e(df_m)

local n = e(N)

local aic = -2 * `ll' + 2 * `df_m'

local bic = -2 * `ll' + log(`n') * `df_m'

display "AIC: " `aic'

display "BIC: " `bic'


*WTP Space

. gen neg_expense = -expense

. mixlogitwtp choice, group(obsid) id(personid) rand(sev1 sev2 sev3 fac1 fac2 sta1 sta2 ratio) price(neg_expense) nrep(500)


*lclogit2  model

lclogit2 choice, rand(sev1 sev2 sev3 fac1 fac2 sta1 sta2 expense ratio) group(obsid) id(personid) nclasses(4) membership(gender age education use) seed(12345678)

display e(bic)

display e(nclasses)

display e(aic)

display e(bic)
```

display e(caic)

display e(ll)

matrix start=e(b)

lclogitml2 choice, rand(sev1 sev2 sev3 fac1 fac2 sta1 sta2 expense ratio) group(obsid) id(personid) nclasses (4) membership(gender age education use) from(start)

*lclogit2 model’s WTP

lclogitwtp2, cost(expense)

4 APPENDIX D: Questionnaire

Preference for home-based healthcare and care services

Home address: the district/county _____ _____ _____ _____ province city street / _____ _____ community/village of villages and towns

Date: ____ _ ____, 2023

Investigator (signed): _____________ Inspector (signed) : _____________

Sequential number: _____________

Dear residents,

Hello! We are a survey team from the School of Medicine and Health Management at Huazhong University of Science and Technology. Our objective is to assess the preferences of community residents for utilizing home-based healthcare and support services. In order to achieve this, we would appreciate your cooperation in providing information about your current status and utilization of services. Please be assured that the survey data will be solely used for academic research purposes, and your responses will be treated with the utmost confidentiality. This questionnaire is expected to take approximately 15 minutes to complete. Kindly respond thoughtfully based on your actual circumstances. Thank you for your valuable participation!

Part 1: Awareness and use of home-based healthcare and support services

Before proceeding with the following questionnaire, we would like to provide a brief introduction to the concept of home-based healthcare and support services. These services pertain to patients who require continuous medical treatment but are unable to care for themselves or face mobility challenges that make it arduous to visit health facilities. These patients rely on on-site medical staff for assistance. Beds are typically installed at their homes or within residential elderly care service institutions. Designated Professionals perform regular checks, administer treatments, and provide care. Additionally, they maintain a comprehensive service record within a specific medical document as part of these healthcare services. Please indicate your situation by typing "✓" in the corresponding box below that aligns with the service description.

**Awareness**	**Unclear**	**Not very clear**	**Average**	**Relatively clear**	**Very clear**
1. Have you heard about home-based healthcare and support services (through news, Wechat public account, etc.)?					
2. Are you aware of the specific contents of home-based healthcare and support services?					
**Trust**	**Very distrustful**	**Low trust**	**Average**	**Relatively trust**	**High**

					**Trust**
3. Your current attitudes to home-based healthcare and support services?					
4. Your attitudes toward home-based healthcare and support services when yourself or a family member has chronic conditions?					
**Utilization**	**Never used**	**Occasionally (less than once every half year)**	**General (almost once every half year)**	**Sometimes (Once every two months.)**	**Often (once every two weeks)**
5. How often have you used home-based healthcare and support services in the past year?					
6. How often has your family used home-based healthcare and support services in the past year?					
**Satisfaction (only those who have used home-based healthcare and support services in the past year)**	**Very dissatisfied**	**Not very satisfied**	**Average**	**Satisfied**	**very satisfied**
7. Are you satisfied with the Insurance reimbursement for home-based healthcare and support services?					
8. Are you satisfied with the current scope of services in home-based healthcare and support services?					
9. Are you satisfied with the attitude of the staff delivering home-					

	based healthcare and support services?	
10.	Are you satisfied with the diagnosis and treatment delivered through home-based healthcare and support services?	

Part 2: Preference for home-based healthcare and support services

Imagine that you are presented with two options for home-based healthcare and support services. These plans vary in terms of service structure, scope of services, service providers, service staff, visiting fee, and insurance reimbursement ratio. Assume that all aspects of the service packages are identical except for the six attributes mentioned. Please select your preferred plan from the 13 scenarios provided below based on your personal preferences. To indicate your choice, type "✓" at the bottom. Your selections will contribute to the enhancement of home-based healthcare and support services and the formulation of related policies. Kindly make your choice after thoughtful consideration.

Note:

Attributes of services in the questionnaire: (1) Health monitoring and management: Health monitoring and management; (2) Diagnosis and treatment: Diagnosis and treatment encompass acute care, sub-acute care, and chronic care management, involving activities such as consultations, examinations, diagnostics, and the provision of medical, nursing, and pharmacy services.

(3) Care support services: Home delivery of meals, assistance with bathing, and the elimination of environmental obstacles to foster independent living.(4) 3 = Mental health services: Psychological counselling , spiritual comfort and palliative care for terminal illness.

The attributes of Institutions in the questionnaire include hospitals, Primary care institutions, and other service institutions. The Professional included in the questionnaire were doctors, nurses and other personnel, and the other personnel included rehabilitation therapists, clinical dietitians and pharmaceutical professionals.

Selection Set 1	Service Pack 1	Service Pack 2
Scope of services	Care support services	Mental health services
Institutions	Primary health care institutions	Hospitals
Health professional	Other personnel	Doctor
Visiting fee	80 yuan per visit	50 yuan per visit
Insurance reimbursement	60%	85%
**I will choose**	**Service Pack 1** ☐	**Service Pack 2** ☐

Select Set 2	Service Pack 1	Service Pack 2
Scope of services	Diagnosis and treatment	Care support services
Institutions	Primary health care institutions	Other service institutions
Health professional	Nurses	Other personnel
Visiting fee	65 yuan per visit	80 yuan per visit
Insurance reimbursement	75%	80%
**I will choose**	**Service Pack 1** ☐	**Service Pack 2** ☐

Select Set 3	Service Pack 1	Service Pack 2
Scope of services	Care support services	Mental health services
Institutions	Other Service institutions	Hospitals

Health professional	Doctor	Nurses
Visiting fee	65 yuan per visit	80 yuan per visit
Medicare reimbursement atio	80%	85%
**I will choose**	**Service Pack 1** ☐	**Service Pack 2** ☐

Select Set 4	Service Pack 1	Service Pack 2
Scope of services	Mental health services	Health monitoring and management
Institutions	Hospitals	Primary health care institutions
Health professional	Other personnel	Doctor
Visiting fee	50 yuan per visit	65 yuan per visit
Insurance reimbursement	75%	80%
**I will choose**	**Service Pack 1** ☐	**Service Pack 2** ☐

Select Set 5	Service Pack 1	Service Pack 2
Scope of services	Diagnosis and treatment services	Care support servicess
Institutions	Primary health care institutions	Other Service institutions
Health professional	Doctor	Nurses
Visiting fee	80 yuan per visit	50 yuan per visit

Insurance reimbursement	85%	75%
**I will choose**	**Service Pack 1** ☐	**Service Pack 2** ☐

Select Set 6	Service Pack 1	Service Pack 2
Scope of services	Health monitoring and management	Diagnostic and treatment
Institutions	Primary health care institutions	Other service institutions
Health professional	Other personnel	Health monitoring and management
Visiting fee	50 yuan per visit	65 yuan per visit
Insurance reimbursement	75%	80%
**I will choose**	**Service Pack 1** ☐	**Service Pack 2** ☐

Select Set 7	Service Pack 1	Service Pack 2
Scope of services	Care support services	Mental health services
Institutions	Hospitals	Primary health care institutions
Health professional	Doctor	Nurses
Visiting fee	50 yuan per visit	65 yuan per visit
Insurance reimbursement	75%	80%
**I will choose**	**Service Pack 1** ☐	**Service Pack 2** ☐

Select Set 8	Service Pack 1	Service Pack 2
Scope of services	Care support services	Mental health services
Institutions	Hospitals	Primary health care institutions
Health professional	Nurses	Other personnel
Visiting fee	80 yuan per visit	50 yuan per visit
Insurance reimbursement	75%	85%
**I will choose**	**Service Pack 1** ☐	**Service Pack 2** ☐

Select Set 9	Service Pack 1	Service Pack 2
Scope of services	Mental health services	Health monitoring and management
Institutions	Primary health care institutions	Other service institutions
Health professional	Doctor	Nurses
Visiting fee	65 yuan per visit	80 yuan per visit
Insurance reimbursement	85%	75%
**I will choose**	**Service Pack 1** ☐	**Service Pack 2** ☐

Select Set 10	Service Pack 1	Service Pack 2
Scope of services	Health monitoring and management	Diagnostic and nursing Services

Institutions	Other Service institutions	Hospitals
Health professional	Doctor	Nurses
Visiting fee	65 yuan per visit	80 yuan per visit
Medical insurance reimbursement ratio	85%	75%
**I will choose**	**Service Pack 1** ☐	**Service Pack 2** ☐

Select Set 11	Service Pack 1	Service Pack 3
Scope of services	Health monitoring and management	Diagnostic and treatment
Institutions	Other Service institutions	Hospitals
Health professional	Other personnel	Doctor
Visiting fee	65 yuan per visit	80 yuan per visit
Insurance reimbursement	75%	80%
**I will choose**	**Service Pack 1** ☐	**Service Pack 2** ☐

Select Set 12	Service Pack 1	Service Pack 3
Scope of services	Health monitoring and management	Diagnosis and treatment service
Service institutions	Hospitals	Primary health care institutions
Health professional	Nurses	Other personnel
Visiting fee	80 yuan per visit	50 yuan per visit

Insurance reimbursement	80%	85%
**I will choose**	**Service Pack 1** ☐	**Service Pack 2** ☐

Choice set 13	Service Pack 1	Service Pack 2
Scope of services	Diagnosis and treatment services	Care support servicess
Service institutions	Primary health care institutions	Other service institutions
Health professional	Nurses	Other personnel
Visiting fee	65 yuan per visit	80 yuan per visit
Insurance reimbursement	75%	80%
**I will choose**	**Service Pack 1** ☐	**Service Pack 2** ☐

Part 3: Basic information

1: Gender	□ Male □ Female
2. Age	Full age of______years
3 Education	□ Middle school and below □ High school or vocational school □ Associate degree □ Bachelor degree □ Master degree □ Doctoral degree
4. Occupation	□ Farmer □ Worker □ Student □ Health worker □ Teacher □Civil Service

	□ Self-employed □ Retired □ Unemployed □ Others
5. Average monthly household income (Yuan)	□≤3000 □3001~5000 □5001~10000 □10001~15000 □15001~20000 □ > 20000
6. Residency	□ Urban □ Urban and rural joint area □ Rural
7. Province	
8. Insurance coverage	□ Basic medical insurance for urban employees □ Basic medical insurance for urban and rural residents □ Commercial insurance □ Other □ No medical insurance
9. I suffer from chronic conditions	□ Yes, what conditions: _____ □ No
10. My family members suffer from chronic conditions	□ Yes, what conditions: _____ □ No
11. Self-rated health	□ Health □ Good □ Fair □ Poor □ Very poor
12. Number of household members in the past year	_____persons

Upon completion of this survey, your responses will hold significant reference value for our academic research. The research team at the School of Medicine and Health Management, Huazhong University of Science and Technology, extends heartfelt gratitude for your valuable participation!

5 APPENDIX D

Table D1. Group Number identified through the latent-class logit model

Group Number	Null Log Likelihood (LL)	BIC	AIC	Class Minimum Share
2	-2219.0416	4547.2003	4476.0832	0.456
3	-2137.7775	4488.0462	4349.5551	0.166
4	-2090.4916	4473.8763	4282.9831	0.085

The null log-likelihood refers to the log-likelihood value of a statistical model assuming the null hypothesis is true. It represents the highest log-likelihood value attainable under the null hypothesis. AIC stands for Akaike Information Criterion, while BIC stands for Bayesian Information Criterion. "Class Minimum Share" indicates a stipulated minimum proportion or percentage of participants that must choose each class or category. This condition ensures that every option or class receives a specific level of consideration or representation during the course of the experiment.
